# Sarcopenia, Dynapenia, and the Impact of Advancing Age on Human Skeletal Muscle Size and Strength; a Quantitative Review

**DOI:** 10.3389/fphys.2012.00260

**Published:** 2012-07-11

**Authors:** W. Kyle Mitchell, John Williams, Philip Atherton, Mike Larvin, John Lund, Marco Narici

**Affiliations:** ^1^Division of Surgery, School of Postgraduate Entry Medicine and Health, University of NottinghamDerby, UK; ^2^Department of Clinical Physiology, School of Postgraduate Entry Medicine and Health, University of NottinghamDerby, UK

**Keywords:** sarcopenia, dynapenia, skeletal muscle, aging, muscle atrophy, muscle aging, muscle quality, strength

## Abstract

Changing demographics make it ever more important to understand the modifiable risk factors for disability and loss of independence with advancing age. For more than two decades there has been increasing interest in the role of *sarcopenia*, the age-related loss of muscle or lean mass, in curtailing active and healthy aging. There is now evidence to suggest that lack of strength, or *dynapenia*, is a more constant factor in compromised wellbeing in old age and it is apparent that the decline in muscle mass and the decline in strength can take quite different trajectories. This demands recognition of the concept of *muscle quality*; that is the force generating per capacity per unit cross-sectional area (CSA). An understanding of the impact of aging on skeletal muscle will require attention to both the changes in muscle size and the changes in muscle quality. The aim of this review is to present current knowledge of the decline in human muscle mass and strength with advancing age and the associated risk to health and survival and to review the underlying changes in muscle characteristics and the etiology of sarcopenia. Cross-sectional studies comparing young (18–45 years) and old (>65 years) samples show dramatic variation based on the technique used and population studied. The median of values of rate of loss reported across studies is 0.47% per year in men and 0.37% per year in women. Longitudinal studies show that in people aged 75 years, muscle mass is lost at a rate of 0.64–0.70% per year in women and 0.80–00.98% per year in men. Strength is lost more rapidly. Longitudinal studies show that at age 75 years, strength is lost at a rate of 3–4% per year in men and 2.5–3% per year in women. Studies that assessed changes in mass and strength in the same sample report a loss of strength 2–5 times faster than loss of mass. Loss of strength is a more consistent risk for disability and death than is loss of muscle mass.

## Introduction

We rely upon skeletal muscle for every interaction with our environment and every activity of daily life. The physical challenges of rising from a chair, dressing and walking, bringing food to the open mouth, chewing and swallowing, clearing respiratory secretions, and managing personal hygiene are taken for granted by most, but these are the very activities which, when compromised due to weakness, necessitate institutional care for a significant proportion of the population. Whilst in some cases a specific cause of weakness such as a neurological disease may be identifiable, an almost inevitable contributing factor will be old age. With old age we see at best compromised physical prowess (Moore, 1975; Meltzer, 1994; Ojanen et al., 2007) and at worse a disabling loss in independence and mobility with approximately a quarter of those over 90 years of age requiring long term residential, nursing, or hospital care in the UK (Office of Fair Trading, 2005; Bajekal et al., 2006). Compromised muscle function has been identified as an independent predictor of hospitalization, disability, and death (Newman et al., 2006).

In this review we outline current understanding of the changes that occur in human skeletal muscles with age. We describe changes in size and changes in function and structure and then describe etiologies and potential interventions.

## Quantitative Changes in Muscle

### Size

Historic observations of the loss of muscle bulk seen in old age, from Aristotle to Shakespeare, have been quoted in recent scholarly works on the topic (Evans, 1995; Narici and Maffulli, 2010). In the last half-century a range of elaborate techniques have confirmed a reduction in size of muscle mass with age. This is especially evident in the comparison of those aged 20–30 years to those over 70 years.

#### Sarcopenia as a concept

This phenomenon was given the name “sarcopenia,” derived from the Greek “sarcos” referring to flesh and “penia,” a lack of, by Rosenberg (1989). The purpose of giving it a title was to strengthen the concept of loss of skeletal muscle with old age, independent of disease process, as an entity, and to stimulate scientific and clinical interest in the area. The term is now in widespread use with thousands of peer-reviewed articles identifying it as a keyword. Whilst originally it referred just to the loss of lean mass, it has also been used to refer to the loss of both strength and size, as discussed below (Morley et al., 2001; Cruz-Jentoft et al., 2010).

It has been suggested that sarcopenia should be considered a “geriatric syndrome” (Cruz-Jentoft et al., 2010). The term “geriatric syndrome” is used to capture those complex but common clinical situations seen in old age, which do not fit into discrete disease categories. Examples include delirium, falls, incontinence, and frailty. Dysfunctions in multiple systems, often at distant sites, contribute synergistically to these syndromes; the relative contributions can be difficult to establish (Inouye et al., 2007). The argument put forward for recognizing sarcopenia as a geriatric syndrome is to promote its identification and treatment even when the exact cause remains unknown.

#### Definition and classification of sarcopenia

No definition of sarcopenia has received universal acceptance. The term was initially used to describe the loss of lean mass with “healthy” aging (Rosenberg, 1989). One widely used definition of sarcopenia proposed in 1998 by Richard Baumgartner was based on a measure of relative muscle mass obtained by dividing absolute muscle mass, evaluated by dual-energy X-ray absorptiometry (DXA), by height squared. In a fashion analogous to the approach used to define underweight, overweight, and obese from BMI, sarcopenia was defined as relative muscle mass lower than two standard deviations below the mean of a large sex-specific reference population aged 18–40 years (Baumgartner et al., 1998). Another definition with a classification of severity, proposed by Ian Janssen in 2002, was based on a skeletal muscle index (SMI) calculated by dividing total muscle mass by total body mass. Muscle mass was evaluated by bioelectrical impedance analysis (BIA). Subjects were considered to have normal SMI if it was within one standard deviation of the sex-specific mean for young adults. Class I sarcopenia was considered present when a subject’s SMI was between one and two standard deviations below the young adult values and class II sarcopenia was present in those subjects more than two standard deviations below the young adult reference (Janssen et al., 2002). This approach was considered comparable to the use of bone mineral density of a young reference group in the classification of normal bone density, osteopenia, and osteoporosis (Kanis, 1994).

Subsequently it has been suggested that the term sarcopenia should also encompass weakness and loss of function (Morley et al., 2001). Rosenberg himself, at a symposium in 1996, declared that the term actually describes important changes in body composition *and* function. The United States National Institutes of Health now recognize this broader definition (National Institutes of Health, 2004). In 2010 the European Working Group on Sarcopenia in Older People published a consensus document which proposed a diagnosis of sarcopenia to require “low muscle mass” accompanied by either “low muscle strength” or “low physical performance.” This group suggested that stages of sarcopenia be recognized; presarcopenia with loss of muscle mass; sarcopenia when this is accompanied by either loss of strength or physical performance; and severe sarcopenia when all three aspects are present (Cruz-Jentoft et al., 2010).

Some reject this use because it implies a proportionality between loss of muscle bulk and loss of strength which, as discussed below, is not the case as with aging the decline in strength exceeds that of muscle size (Narici and Maffulli, 2010). The term *dynapenia* has been proposed to refer to the functional compromise of the entire neuromuscular apparatus (Clark and Manini, 2008) and although there is good evidence that this concept is of clinical significance (Clark and Manini, 2010) the term is yet to achieve widespread usage. Some writers argue against the separation of dynapenia and sarcopenia due to the risk of nomenclature introducing confusion (Cruz-Jentoft et al., 2010).

It has also been proposed that the term sarcopenia need not be reserved for muscle loss with old age and that the phenomenon may be seen, albeit less frequently, in the young; as is the case with dementia or osteoporosis. Similarly, sometimes the term is used to encompass any kind of muscle loss even that caused by a single identifiable disease (“secondary sarcopenia” as opposed to “primary sarcopenia” in an otherwise well individual; Cruz-Jentoft et al., 2010). Other writers reserve the use of sarcopenia for its origin use, considering secondary sarcopenia to be an aspect of cachexia (Thomas, 2007). A global consensus on the use of the term sarcopenia has not yet been achieved.

#### Clinical impact of sarcopenia

Two decades ago when the concept of sarcopenia entered vogue it was envisaged that loss of muscle mass was the major determinant of the decline in physical function with age and a modifiable risk factor in disease and disability (Frontera et al., 1991; Evans, 1995). Sarcopenia, as described above by Baumgartner, was independently associated with use of a frame or walker, with falls, and, in both sexes, with physical disability even when adjusted for age, obesity, and comorbidities (Baumgartner et al., 1998). Janssen’s definition of sarcopenia was also used to associate low muscle mass with functional impairment and disability (Janssen et al., 2002). In the ilSIRENTE study, individuals in the low tertile of mid-arm muscle circumference, a simple anthropometric index of muscle mass, had a significantly greater mortality than individuals in the high tertile (Landi et al., 2005).

However other data do not support the strong association between low fat free or muscle mass and disability and death. Low fat free mass failed to demonstrate association with self-reported physical disability in both the Cardiovascular Health Study (Visser et al., 1998b) and the Framingham Heart Study (Visser et al., 1998a). The HABC Study has collected prospective body composition, strength, function, and health and survival data for more than 3000 older people over several years. It failed to demonstrate increased compromise in lower limb function in sarcopenic men when sarcopenia was defined as low appendicular lean mass/height^2^ (Delmonico et al., 2007). In this study the incidence of mobility limitation does increase with low muscle size (mid-thigh muscle cross-sectional area; CSA by CT) and low muscle strength (maximal isokinetic knee extension strength) but after muscle strength was taken into account, muscle area did not remain a significant factor associated with incident mobility limitations. This suggests muscle strength mediates any relationship between muscle mass and mobility limitation (Visser et al., 2005). Moreover, this study did not show upper or lower limb muscle mass to be associated with mortality (Newman et al., 2006).

Lean mass, measured by DXA, and thigh muscle CSA, measured by CT, in 3011 adults aged 70–80 years failed to show association with hospitalization rates in the subsequent 4.7 years (Cawthon et al., 2009).

#### Epidemiology of sarcopenia

Using the definition described above; muscle mass measure by DXA/height^2^ less than an index of two standard deviations below the mean of a sex matched young reference population; the NMEHS study showed that in a New Mexico population c. 15% of males and c. 24% of females aged 65–70 years were sarcopenic. This rose to >50% in both sexes among the over 80s. Sarcopenia was generally more prevalent in Hispanics than non-Hispanic whites (Baumgartner et al., 1998). When similarly measured in a Caucasian New England population 53% of men and 31% of women aged over 80 years were sarcopenic (Iannuzzi-Sucich et al., 2002). A much lower prevalence of sarcopenia was seen amongst Danish women with sarcopenia diagnosed in 12% of >70-year olds (Tanko et al., 2002) and in Taiwan, with 26% of men and 19% of women over 80 sarcopenic (Chien et al., 2008).

According to the definition of Janssen et al. (2002) described above, in a study using a nationally representative cohort of Americans, 50% of men and 72% of women over 80 years were sarcopenic; with 7 and 11% suffering Class II sarcopenia.

#### Quantifying age-related changes in muscle mass

Numerous studies have aimed at quantifying the decrement in skeletal muscle bulk, either volume or mass. Attempts have been made to mathematically describe the decline in function of the musculoskeletal system seen with advancing years as if decline is a uniform process that starts at completion of growth (Sehl, 2001). However there is little consensus on the rate of decline. Estimates of loss of muscle mass by age 18–80 years range from 8 to 49% (Novak, 1972; Tzankoff and Norris, 1977). Studies done over the last five decades are summarized in Tables 1– 3. Table 1 summarizes those studies that compare cohorts in or near peak muscle bulk to those in their seventh, eighth, or ninth decades (Lexell et al., 1983; Young et al., 1985). An approximation of percentage loss per decade has been calculated for each study. The median value reported across studies for the rate of loss in men is 4.7% of peak mass per decade and in women it is 3.7% per decade. Table 2 summarizes those studies that specifically look at rate of loss after the seventh decade (Baumgartner et al., 1995). In many studies a failure to report actual age characteristics of groups prevents calculation of loss per decade.

**Table 1:** **Summary of cross-sectional studies of changing skeletal muscle mass, comparing those in age groups considered to represent “peak muscle mass” versus elderly**.

Study	Technique	Estimate	Sex	Young (years)	Aged (years)	*n*	Change	% Change	% Change/year
Novak (1972)	Total body potassium	Fat free mass	M	18–25	65–85	27, 18	−13 kg	−22	−0.44
		Cell mass					−7.3 kg	−22	−0.44
		Fat free mass	F			89, 13	−3.0 kg	−8.0	−0.16
		Cell mass					−1.7 kg	−8.0	−0.16
Tzankoff and Norris (1977)	24 h urinary creatinine excretion	Muscle mass	M	20	90	14, 12	−965 mg/24 h	−49	−0.7	
				20	80	14, 103	−745mg/24 h	−38	−0.63
Cohn et al. (1980)	Total body nitrogen (Prompt gamma neutron-activation technique)	Fat free mass	M	20–29	70–79	24, 9	−9.0 kg	−14	−0.28
		SMM					−10.9 kg	−45	−0.9
		Fat free mass	F	20–29	70–79	10, 8	−7.8 kg	−18	−0.36
		SMM					−4.0 kg	−40	−0.8
Borkan et al. (1983)	Computed tomography scan	Upper leg muscle CSA	M	46.3 ± 2.6	69.4 ± 4.1	21, 20	−18.2 cm^2^	−12	−0.52
		Upper arm muscle CSA					−6.4 cm^2^	−11	−0.48
	Total body potassium	Fat free mass					−6.6 kg	−11	−0.48
Lexell et al. (1983)	Cadaveric dissection	Vastus lateralis CSA	M	30 ± 6	72 ± 2	6, 6	−576 mm^2^	−17.6	−0.42
Young et al. (1985)	Ultrasound scan	CSA of quadriceps muscles (mid-thigh)	M	20–30	70–80	12, 12		−25	−0.5
Lexell et al. (1988)	Cadaveric dissection	Vastus lateralis CSA	M	19 ± 3	73 ± 2	9, 9	−960 mm^2^	−26	−0.48
				19 ± 3	82 ± 1	9, 8	−1584 mm^2^	−43	−0.68
Janssen et al. (2000)	Magnetic resonance imaging	SMM	M	18–29	>70	66, 11	−5.9 kg	−18	−0.36
		Lower body SMM					−4.7 kg	−25	−0.5
		Upper body SMM					−0.8 kg	−5.6	−0.11
		SMM	F	18–29	>70	40, 19	−3.8 kg	−17	−0.34
		Lower body SMM					−2.8 kg	−22	−0.44
		Upper body SMM					−1.0 kg	−11	−0.22
Kyle et al. (2001)	Dual-energy X-ray absorptiometry	ASMM	M	18–34	>80	68, 26	−5.4 kg	−19.9%	−3.3
			F	18–34	>80	40, 30	−2.6 kg	−14.1%	−2.3
Silva et al. (2009)	Dual-energy X-ray absorptiometry	SMM	M	18–80 mean 40 ± 14.4		468	−1.58 kg/decade after 27	N/A	−0.46
			F	18–80 mean 44.5 ± 15.9		1280	−0.81 kg/decade after 27	N/A	−0.46
Wroblewski et al. (2011)	Air displacement plethysmography and MRI in high-level recreational athletes	SMM	M	44.8 ± 3.2	65.4 ± 2.2	5, 5	−4.1 kg	−6.7	−0.32
				44.8 ± 3.2	76.3 ± 3.3	5, 5	−7.3 kg	−12	−0.38
			F	47.0 ± 2.8	65.0 ± 3.0	5, 5	−4.3 kg	−9.8	−0.54
				47.0 ± 2.8	74.8 ± 3.7	5, 5	−7.0 kg	−16	−0.57

**Table 2:** **Summary of cross-sectional studies of changing skeletal muscle mass, comparing between groups within the aged population**.

Study	Technique	Estimate	Sex	Young (years)	Aged (years)	*n*	Change	% Change	% Change/year
Novak (1972)	Total body potassium	Fat free mass	M	55–65	65–85	42, 18	−6.5 kg	−12	
		Cell mass		55–65	65–85	42, 18	−3.6 kg	−12	
		Fat free mass	F	55–65	65–85	54, 13	−0.8 kg	−2	
		Cell mass		55–65	65–85	54, 13	−0.4 kg	−2	
Tzankoff and Norris (1977)	24 h urinary creatinine excretion	Muscle mass	M	80	90	103, 12	−220 mg/24 h	−18	−1.8
Cohn et al. (1980)	Total body nitrogen (prompt gamma neutron-activation technique)	SMM	M	60–69	70–79	10, 9	−4 kg	−23	
			F	60–69	70–79	14, 8	−0.9 kg	−13	
Lexell et al. (1988)	Cadaveric dissection	Vastus lateralis CSA	M	73 ± 3	82 ± 1	9, 8	−624 mm^2^	−23	−2.6
Frontera et al. (1991)	Hydrostatic weighing	Fat free mass	M	50.5 ± 2.8	68.5 ± 2.8	24, 34	−4.8 kg	−8.0	−0.43
			F	50.2 ± 2.6	69.0 ± 3.8	28, 34	−4.3 kg	−11	−0.58
	24 h urinary creatinine excretion	Muscle mass	M	50.5 ± 2.8	68.5 ± 2.8	17, 29	−2.7 kg	−9.7	−0.52
			F	50.2 ± 2.6	69.0 ± 3.8	19, 20	−3.5 kg	−19	−1
Baumgartner et al. (1995)	Dual-energy X-ray absorptiometry	Appendicular SMM	M	60–70	>80	17, 32	−2.9 kg	−12	
			F	60–70	>80	50, 56	−1.6 kg	−10	
		Fat free mass	M	60–70	>80	17, 32	−4.8 kg	−8.2	
			F	60–70	>80	50, 56	−2.7 kg	−6.8	
	Anthropometrics	Bone free mid-arm muscle CSA	M	60–70	>80	17, 32	−7.0 cm^2^	−13	
			F	60–70	>80	50, 56	+0.6 cm^2^	+1.7	
Janssen et al. (2000)	Magnetic resonance imaging	SMM	M	60–69	>70	14, 11	−0.4 kg	−2.2	
		Lower body SMM		60–69	>70	14, 11	−	−25	
		Upper body SMM		60–69	>70	14, 11	−	−5.6	
		SMM	F	60–69	>70	11, 19	−0.4 kg	−2.2	
		Lower body SMM		60–69	>70	11, 19	−0.8 kg	−7.6	
		Upper body SMM		60–69	>70	11, 19	+0.2 kg	+2.7	
Kyle et al. (2001)	Dual-energy X-ray absorptiometry		M	60–69	70–79	25, 40	−0.5 kg	−2.1	
				70–79	>80	40, 26	−1.7 kg	−7.2	
			F	60–69	70–79	22, 48	−0.3 kg	−1.8	
				70–79	>80	48, 30	−0.7 kg	−4.2	
Wroblewski et al. (2011)	Air displacement plethysmography and MRI in high-level recreational athletes	SMM	M	65.4 ± 2.2	76.3 ± 3.3	5, 5	−3.2 kg	−5.6	−0.51
			F	65.0 ± 3.0	74.8 ± 3.7	5, 5	−2.7 kg	−6.9	−0.7

**Table 3:** **Summary of longitudinal studies of change in human muscle mass**.

Study	Technique	Estimate	Sex	Baseline (years)	FU (years)	*n*	Change	% Change	% Change/year
Frontera et al. (2000)	Computed tomography	Thigh CSA	M	65.4	12.2	7	−24.4 cm^2^	−12.5	−1.0
		Thigh muscle CSA					−19.8 cm^2^	−14.7	−1.2
		Thigh extensor CSA					−10.3 cm^2^	−16.1	−1.3
		Thigh flexor CSA					−5.2 cm^2^	−14.9	−1.2
Hughes et al. (2002)	Hydro-densiometry	Fat free mass	M	61.1	9.5	53	−1.1 kg	−1.9	−0.2
			F	60.0	9.9	78	−0.1 kg	−0.24	−0.024
Dey et al. (2009)	Bioelectrical impedance	Fat free mass	M	70	5	38	−2.02 kg	−3.6	−0.18
(Delmonico et al., 2009) HABC	Computed tomography	Thigh muscle CSA	M	73.6	5	813	−6.8 cm^2^	−4.9	−0.98
			F	73.2	5	865	−3.2 cm^2^	−3.2	−0.64
			F	70	5	49	−0.93 kg	−2.1	−0.16
(Koster et al., 2011) HABC	DXA	Lean leg mass	M	74.2	7	1129	−1.02 kg	−5.6	−0.8
			F	73.9	7	1178	−0.62 kg	−4.9	−0.07

Some studies demonstrate an ever-accelerating wastage with the rate of loss expressed as a factor of age^2^ (Kehayias et al., 1997; Janssen et al., 2000). Others describe a linear loss in later years following a plateau or muscle gaining phase (Gallagher et al., 1997; Kyle et al., 2001; Silva et al., 2009). A large study with a robust technique for measuring summed four-limb (appendicular) skeletal muscle mass (DXA, see below) has shown it to be almost static from ages 18 to 60 years, with a slight gain of muscle throughout this period in men and a slight loss in women (Kyle et al., 2001). The age at which decline starts has been reported as 27 years (Silva et al., 2009), 45 years (Janssen et al., 2000), and 60 years (Kyle et al., 2001).

Several reasons contribute to the differences between studies. Diverse techniques are employed to provide estimate skeletal muscle mass. Fat free mass or fat free cell mass have been used as indices of muscle mass (Novak, 1972; Cohn et al., 1980; Borkan et al., 1983). Total body potassium (TBK) and nitrogen (TBN) estimations of fat free mass may overestimate losses as K^+^/gram skeletal muscle itself, a measure assumed constant in these studies, has been shown to decrease with age (Kehayias et al., 1997). Similarly, creatinine excretion per gram of muscle has been shown to be lower in old men compared to young yet the assumption of lifelong constant excretion rates has been used in the calculation of muscle mass estimates in non-agenarians (Tzankoff and Norris, 1977). Non-invasive imaging techniques (computed tomography; CT and magnetic resonance; MRI) are now regarded as the gold standard in measurement of whole body and limb specific muscle mass and these modalities estimate a loss of peak muscle mass of around 20% even in those aged 70–88 years (Janssen et al., 2000). In the last two decades DXA has been widely used in assessing body composition including measurement of lean tissue mass. It can provide an estimate of appendicular and total skeletal muscle mass which correlates closely to that measured by CT but at a much lower cost and exposure to a fraction of the ionizing radiation (Wang et al., 1996).

All cross-sectional studies make the assumptions that a contemporary young sample of the population is a fair proxy for their aged sample at a time in the past. Such studies are influenced by secular changes, i.e., intergenerational differences representing changes in the population characteristics rather than age-related changes that would be evident within a cohort over time.

It could be argued that some cross-sectional studies are particularly vulnerable to differences in selection criteria between young and old. For example studies looking at cadavers of those who died as a result of trauma assume that amount of muscle mass *per se* does not influence the probability of a traumatic death. If, indeed, larger muscle mass in young men was a risk factor *for* traumatic death (for example, due to association with manual laboring employment) whilst larger muscle mass *reduced* the chance of traumatic death in old age (for example, protective against falls), then a selection bias would contribute to the marked loss seen in such studies (Lexell et al., 1988).

**Figure 1:**
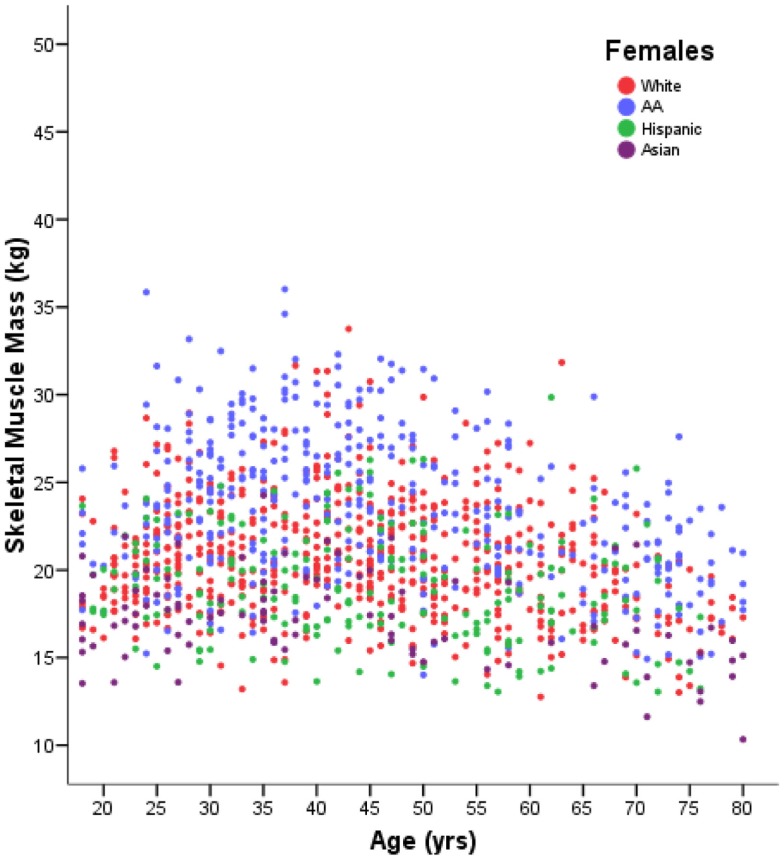
**Muscle mass in 1280 females aged 18–80 years, measured by DXA**. With permission Z. M. Wang and A. M. Silva, adapted from Silva et al. (2009).

Whilst all cross-sectional studies are at risk of these systematic influences, large studies with robust techniques will provide a good measure of variability between individuals in each age group as well as giving an estimate of changes with age. Figure 1 demonstrates the subtlety of the downward trend in muscle mass with aging when it is seen in the context of the high degree of variability between individuals. This concept has not previously been highlighted in reviews of the subject.

#### Age-related changes are not uniform across the body

The rate of loss is not uniform across muscles. In an MRI-based study of 200 women and 268 men, the rate of loss of lower limb muscle was more than twice the rate of loss of upper limb muscle (Janssen et al., 2000), supporting previous evidence from CT (Borkan et al., 1983) and DXA (Gallagher et al., 1997) based measurements.

#### Sex, race, and menopausal status may impact on age-related changes in muscle mass

At any given age males possess more muscle bulk than females, even after correction for height and weight. Men exhibit larger age-related decreases in muscle mass compared to women (Gallagher et al., 1997). However this difference all but disappears when loss is expressed as a proportion of peak muscle mass (Janssen et al., 2000; Silva et al., 2009).

It has been proposed that menopausal status has a significant impact upon the maintenance of muscle mass and that transition into the menopause has been associated with a reduction in muscle mass (Sirola and Kroger, 2011). However the evidence to support this claim has been equivocal. The first study proposing an accelerated loss of muscle at menopause used TBK to measure cell mass and demonstrated a negligible rate of loss in pre-menopause and a significant inverse correlation with age amongst post-menopausal women (Aloia et al., 1991). Another study used both the TBK technique and also DXA to measure appendicular muscle mass in both men and women and the data displayed very similar trends in both sexes, with different regression models being requires to describe the relationship between muscle mass and age before and after age 60 years (Kyle et al., 2001). Thus it may be argued that the increased loss of muscle seen after menopause is more age-related than menopause related. This is supported by another study that shows leg lean mass to be inversely correlated with age but not menopausal status (Douchi et al., 2002).

Although men loose more muscle with aging, in absolute and relative terms, it seems that women suffer more from the consequences of lean tissue mass (Janssen et al., 2002), perhaps due to their lower starting mass and their greater longevity.

Studies have attempted to quantify separately the age-related changes in muscle bulk in different ethnic groups living in the same area. African American men and women have been shown to have higher peak muscle mass than Whites but experience greater absolute and relative age-related losses (Gallagher et al., 1997; Silva et al., 2009).

The rate of loss in high-level recreational athletes, who continue to train 4–5 times a week, was similar to subjects not selected according to athletic activity, albeit happening against a different baseline (Frontera et al., 1991; Wroblewski et al., 2011).

#### Limitations of cross-sectional observation

These cross-sectional studies provide a measure of muscle mass across ages at a moment in time. They cannot support the assumption that individuals follow trajectories of change calculated from arithmetic lines of best fit. Longitudinal studies, with all the associated difficulties in logistical aspects of execution, are the only way to assess trends in individuals.

Table 3 summarizes the few longitudinal studies that assess estimates of muscle mass over time. Given the huge size of the overlapping sample considered by both Koster et al. (2011), Delmonico et al. (2009) who used data from the health, aging and body composition (HABC) study, it should be recognized that their findings; an annual loss of 0.8–0.98 and 0.64–0.7% of leg lean mass per year throughout the eight decade, in men and women, respectively; are the most reliable to date for a population of initially well functioning community dwelling men and women. There is marked discrepancy with the results of the previous largest longitudinal study of Hughes et al. (2002), who considered total lean mass measured by underwater weighing and reported an annual loss of 0.2% in men and approximate stability in women throughout the seventh decade. Contributing to this discrepancy may be the older age of the HABC subjects; the method of recruitment, with HABC subjects being randomly selected Medicare beneficiaries whilst Hughes followed up patients responding to newspaper advertisements; and the asymmetrical loss with lower limb muscle atrophy exceeding upper limb. However these studies consistently report losses of less than 1% per year. Despite this a figure of 1–2% loss per year over 50 is widely quoted and misattributed to Hughes et al. (Thomas, 2007; Rolland et al., 2008; Peake et al., 2010; Sirola and Kroger, 2011).

#### Age-related loss of muscle is only a part of age-related change in body composition

Against a period of almost-constant muscle mass lasting up to four decades, most individuals gain total body weight due to an increase in fat mass (Kyle et al., 2001). Thus a reduction in relative muscle mass, expressed as a fraction of body weight, can be seen to drop from the third decade of life (Janssen et al., 2000). Most people continue to gain fat mass in the eighth decade when muscle mass is decreasing (Goodpaster et al., 2006).

Muscle mass does decrease with advancing age. On average, in every age group, men carry more muscle mass than women. Men loose more muscle both in absolute terms and a proportion of total. Historical methods of estimating changes in muscle mass have tended to overestimate loss. Throughout much of adult life, muscle mass remains fairly constant. Average rates of loss in those aged over 70 years are in the region of 0.5–1% per year. Most individuals aged over 70 years will possess about 80% of the muscle mass of those aged 20–30 years. There is great variability between individuals at any given age. Sarcopenia is a term that is widely used to describe the phenomenon of muscle loss with old age. There is still much debate surrounding its exact definition. The prevalence of sarcopenia varies with geography, ethnicity, and definition. It may affect half of octogenarians. Some studies show low muscle mass to be a risk factor for disability and death. The health impact of low muscle mass is not as consistent at the impact of low strength.

### Function

Most skeletal muscles act through tendons to move joints against resistance. The force developed depends upon the number of sarcomeres acting in parallel and the force per sarcomere as well the mechanical advantage at which the muscle works. The maximum force that can be applied against an immovable object is the *isometric strength*. *Isokinetic strength* describes the peak torque that can be developed against a load that moves through an arc at a fixed angular velocity. *Power* describes the rate of energy transfer and is therefore the product of velocity and force of contraction. The velocity of shortening will depend upon the number of sarcomeres in series as well as the speed of shortening of each sarcomere. Concentric contractions occur within a shortening muscle that acts against a load which it overcomes; eccentric contractions occur when the muscle is actively lengthened by external forces. Thus intrinsic stiffness of a muscle will hinder force generation during concentric contractions but contribute to force generation during eccentric contractions. *Fatigability* describes the exercise-induced reduction in the ability to exert muscle force or power. As most muscles act across joints via tendons, changes in other connective tissue elements will indirectly affect the muscle’s mechanical behavior (its length–force relation) and impact upon physical performance as will sensorimotor and cognitive processes such as balance, attention, and motivation (Narici and Maganaris, 2007). For these reasons assessing, interpreting, and describing changes in muscle function with age is much more challenging than changes in size of muscle mass, which can be expressed relatively easily as an absolute or relative change in mass or as a simple index thereof, such as muscle mass/height^2^ or % body mass.

#### Hand grip strength and upper limb isometric strength

The use of simple hand grip strength near-isometric dynamometers has been established for more than seven decades (Fischer and Birren, 1946). These permitted an early quantification of changing muscle function with age and also raised awareness of the tendency for cross-sectional studies to underestimate each individual’s rate of decline because of relatively fewer weak individuals surviving to be represented in the older samples (Clement, 1974). The NORA cohort of initially healthy, independent Scandinavian 75-year-old men and women showed a drop in grip strength in 5 years of 20 and 15%, respectively. This was contrasted to a loss of fat free mass as measured by BIA of 3.6 and 2.1% (Dey et al., 2009) suggesting a dissociation between loss of muscle size and strength.

Hand grip dynamometers can be portable and inexpensive and therefore can easily be introduced into clinical practice. Low hand grip strength predicts disability, hospitalization, and mortality. Among a sample of circa 2500 independent living Mexican Americans over 65 years followed up for 7 years, incident disability was more common in the lowest quartile compared to the highest hand grip strength quartile even after adjustment for confounding factors (HR 1.9, 95% CI 1.14–3.17 in men and 2.28, 95% CI 1.59–3.27 in women; Al Snih et al., 2004). The Leiden 85-plus Study looked at Dutch individuals over 85 years and showed low handgrip strength to be a predictor of accelerated decline in activities of daily living (ADL)-ability and cognition (Taekema et al., 2010). Hospitalization was more common in those aged 70–80 years and in the lowest quartile of hand grip strength compared to the highest (OR 1.47, 95% CI 1.3–1.78; Cawthon et al., 2009). Among 463 Finnish people aged 75–84 years participating in the EVERGREEN project, the risk of death within 4 years was higher in those with grip strength below the sample mean (OR = 1.86, 95% CI 1.13–3.07; Laukkanen et al., 1995). Progressive increases in mortality amongst men aged >60 years have been observed between quartiles when ranked by hand grip, an effect that remained significant even after adjustment for total muscle mass as calculated by 24 h creatinine excretion (Metter et al., 2002). The HABC Study showed mortality to be associated with hand grip strength and this effect remained significant even after adjustment for arm lean tissue mass as measured by DXA (Newman et al., 2006). The Leiden 85-plus Study showed the increased mortality associated with low hand grip strength persists as far as the end of the ninth decade (Ling et al., 2010). These findings suggest that measurement of hand grip strength may have a role in clinical assessment and risk stratification in the elderly.

#### Knee extensor strength and lower limb isokinetic strength

Assessment of knee extensor isokinetic strength, or peak torque at a fixed angular velocity, allows a dynamic measure of lower limb function. Lower limb strength is lost more rapidly than upper limb strength (Frontera et al., 2000), in line with the loss of lean mass described above. However the loss in strength greatly exceeds the loss of muscle mass. Among 1678 older people followed for 5 years, men lost c. 16% of knee extensor strength but only 5% of thigh muscle mass whilst women lost 13% of strength but only 3% of mass. In those patients with stable body weight or who lost weight, the loss of strength exceeded the loss of muscle mass two- to fivefold. Individuals who gained body weight still lost strength despite an increase in muscle mass. Loss of muscle mass only accounted for a small fraction of the between subject variability in the loss of knee extensor strength (6 and 8% in men and women, respectively). This reinforces the notion of a clear dissociation between loss of muscle and loss of strength (Hughes et al., 2002; Delmonico et al., 2009).

Patients with low knee extension strength are at increased risk of disability and death. The HABC Study shows an increase in incident disability in the lowest compared to highest quartile by quadriceps strength (OR 2.02, 95% CI 1.39–2.94; Visser et al., 2005). The same study shows increased mortality by quadriceps strength per standard deviation of 48 Nm, with a crude hazard ration of 1.51 (95% CI 1.28–1.79) in men and 1.65 (95% CI 1.19–2.3) in women (Newman et al., 2006). The EVERGREEN project showed the risk of death within 4 years was higher in those with knee extension strength below the sample mean (OR = 2.52, 95% CI 1.50–4.42), a higher mortality risk than that associated with low hand grip strength in the same sample (Laukkanen et al., 1995).

#### The preservation of eccentric strength with aging

The evidence presented above shows compromise in strength of isometric contractions, when the muscle is held at a fixed fiber length during activity and in dynamic, concentric contractions when the muscle shortens during the generation of force. When peak torque is measure in eccentric contractions there is a relative preservation of strength in old age and other chronic disease states associated with muscle loss and weakness (Porter et al., 1997; Phillips et al., 1998; Klass et al., 2005). It has been proposed that the accumulation of non-contractile material within the muscle increasing passive stiffness and changes in the contractile properties of muscle fibers resulting in “active stiffness” both contribute to this phenomenon (Roig et al., 2010).

#### Loss of peak torque depends upon angular velocity

During concentric contractions the peak torque developed depends upon the angular velocity of the load. The loss of peak torque with aging is greater at higher angular velocities (Yu et al., 2007). In young men, the ratio of elbow flexion peak torque developed at 240 versus 60 s^−1^ is about 0.9. In elderly men this has been shown to drop to c. 0.5 (Pousson et al., 2001).

#### Loss of power

Power describes a muscle’s ability to do work and is thus the rate of transfer energy. It is equal to the product of the force developed and the velocity of contraction. Power is lost faster than strength (Skelton et al., 1994; Izquierdo et al., 1999). Measuring and comparing power is much more difficult than measuring strength. The reasons for this are discussed in a review of the topic (Macaluso and De Vito, 2004).

#### Fatigability

Anecdotal evidence may suggest *fatigue* constitutes an element of normal aging. However the observation of increased rapidity of tiring upon repeated performance of the same task reflects the age-related decrease in strength, outlined above, more than fatigability of muscles. As maximum strength decreases, and body mass increases or stays relatively constant, the fraction of maximum force needed to perform the same physical task, e.g., stair-climb or rise from chair, will increase. Fatigue occurs more rapidly with increasing task intensity and the maximum endurance time decreases in an non-linearly fashion as task intensity increase (Frey Law and Avin, 2010).

However the physiological phenomenon of *muscle fatigue*, which has been defined as “an exercise-induced reduction in the ability to exert muscle force or power” (Bigland-Ritchie and Woods, 1984) would require that comparisons between age groups be based upon performance at a constant task intensity (% of maximal force). A recent meta-analysis included data from 46 publications which reported fatigue tasks (voluntary activation) performed at relative intensity in both young (18–45 years) and old (>55 years). This work demonstrated significant fatigue resistance in the aged. Subgroup analysis by task showed significant fatigue resistance when tasks involved sustained or intermittent isometric tasks but this effect was lost when tasks were dynamic (Avin and Law, 2011). This is in keeping with studies that have showed an accelerated loss in Type II fibers (Larsson and Karlsson, 1978; Lexell et al., 1988).

#### Individual fiber observations

The *in vivo* human observations described above will reflect not only factors intrinsic to muscle fibers but will also be influenced by differences in intramuscular fiber orientation, differences in the mechanical leverage provided by the bony anatomy of joints, the elasticity of tendons, the pattern of motor unit recruitment, and the activation of antagonist muscles. Even multicellular muscle preparations, independent of these variables, will depend upon interpreting the contribution of different fiber types. The study of chemically skinned, single fibers allows the examination of myofilament behavior in a near physiological environment yet without the confounding effects of intercellular connective tissue.

It has thus been demonstrated that with aging, the maximum shortening velocity of type I and IIA fibers decreases by c. 20–46 and 10–30%, respectively, and these changes are seen in both males and females (Larsson et al., 1997; Yu et al., 2007). The small numbers of mixed type and type IIx fibers and the variability between fibers make assessment of these types difficult but a trend toward slowing is seen in all fiber types (D’Antona et al., 2003).

Consistent with a decrease in maximum shortening velocity of each fiber type is a decrease in the actin sliding velocity on purified myosin isoforms prepared from aged muscle determined by *in vitro* motility assays (Hook et al., 2001; D’Antona et al., 2003).

The specific tension, or force per unit CSA, has also been shown to decrease with age in both sexes; by c. 16–33% in type I, 14–25% in type IIA, and possibly up to 50% in type IIx (Larsson et al., 1997; D’Antona et al., 2003; Yu et al., 2007).

Within fibers, myosin concentration falls with age. Within each fiber type, the specific tension generated is almost proportional to the myosin concentration observed suggesting that the loss of specific tension is the result of dropping myosin concentrations (D’Antona et al., 2003).

Strength decreases with advancing age. Average rates of loss are 2–4% per year. This is 2–5 times faster than muscle mass is lost. Low strength, both assessed by hand grip and knee extension, predicts disability and death. Factors other than loss of mass account for much of the observed loss of strength. Age compromises the ability to generate torque more at high than low angular velocities. Power is lost more quickly than strength. Relative preservation of eccentric strength and fatigue resistance and is a feature of aged muscles. These phenomena are seen at a single fiber level. Underlying the observed loss of whole muscle function is a slowing of actin-myosin interaction and a reduction in myosin concentration.

## Qualitative Changes in Muscle with Advancing Age

Human skeletal muscle consists of fibers or myofibers, individual multinucleated terminally differentiated cells, usually 20–80 μm in diameter and up to many centimeters long. Fibers are sheathed in insulating endomesium. Nuclei are fixed in a non-random distribution throughout within each fiber, each nucleus regulating protein synthesis within a volume of cytoplasm, its myonuclear domain (MND). Muscle fibers may contain many thousand nuclei; for example in human vastus lateralis each fiber contains about 100 nuclei per millimeter length (Cristea et al., 2010). Arranged along the surface of each muscle fiber are satellite cells which function as a stem cell population; satellite cell mitosis can replenish myonuclear number and producing new satellite cells throughout life.

Running along the length of each fiber are myofibrils; the ultastructural elements responsible for force generation. Each myofibril is composed chiefly of interdigitating actin and myosin myofilaments. In a fiber from a large muscle like gastrocnemius, there may be 1000 myofibrils each composed of c. 1500 myosin and 3000 actin myofilament.

The alignment of interdigitating actin and myosin gives skeletal muscle its striated appearance on microscopy. A sarcomere describes an individual repeated unit of the myofilament and in humans it is about 2 μm long and so 5000 sarcomeres lie in series along each centimeter of muscle fiber. Groups of a few to a few hundred fibers are arranged into fascicles, each just visible to the naked eye and bound together with a connective tissue sheath, the perimesium.

The length of each fascicle will determine the number of sarcomeres in series and therefore the maximum velocity with which it contracts. The CSA of fibers will dictate the number of sarcomeres contracting in parallel and therefore the maximum force generated. Most muscles involved in locomotion are pennate; that is to say the long axis of each fascicle lies at an angle to the axis of traction. Pennation allows more fibers to act in parallel and with hypertrophy, the angle of pennation increases. Thus a physiological CSA of a muscle, perpendicular to the long axis of the fibers, can greatly exceed its anatomical CSA. The volume of a muscle will be the product of its physiological CSA and the fascicle length.

Alpha-motoneurons are the large lower motor neurons that form synapses on muscle fibers. Their cell bodies lie in the ventral horn of spinal gray matter. Each innervates a variable number of muscle fibers which, together with the nerve, constitute a motor unit. Average motor unit size ranges from less than three fibers in the case of the extraocular muscles, c. 180 in the case of soleus to >2000 fibers in the gastrocnemius. The total number of motor units making up some human muscles has also been quantified. This is highly variable between people. In one young sample the range in the number of motor units making up biceps brachi was 58–190 and in the median innervated thenar muscles was 102–421. As described below this changes with age. Due to the security of transmission at the neuromuscular junction between nerve and muscle it is normal for all muscle fibers in a motor unit to fire together making the motor unit the smallest increment of muscle recruitment.

Muscle fibers can be classified according to their predominant myosin heavy chain (MyHC) isoform expression. Type I fibers express MyHC I and demonstrate slow contractile velocity with numerous mitochondria and plentiful myoglobin and hence significant oxidative capacity. Type II fibers are subdivided type IIA and IIx (previously called IIB). Fibers expressing MyHC IIA are packed with more contractile elements each of which contracts faster and generates more force than type I but have fewer mitochondria, less myoglobin, and so less oxidative capacity and therefore less capacity for sustained force generation. Fibers expressing MyHC IIx contract more vigorously than type IIA fibers but with even lower aerobic capacity and are therefore more reliant upon glycolytic metabolism. Each motor unit is composed of fibers of the same type. Type I motor units consist of fewer fibers than Type IIx. Myosin isoform expression is dynamic and can change with training, immobility, or disease (Goldspink, 1985; Guyton, 1991; Purves et al., 2001; Narici et al., 2003; Davies Re and Gergely, 2012).

### Changes in muscle architecture

The architecture of muscle describes the three-dimensional arrangement of its component fibers and is a major determinant of its force and excursion capability (Lieber and Friden, 2001). Muscle architecture is dynamic and changes with hypertrophy. Specific age-related change have also been described using ultrasound to measure fascicle length and pennation angle. For example, in the pennate plantar flexor gastrocnemius medialis, it has been shown that the decreases in volume comparing old to young men, measured at 24–31% by CT and MRI, were due not just to fewer, thinner fibers but also in part due to *shorter* fibers; fascicle length dropped by 10–20% (Narici et al., 2003; Thom et al., 2007). This will contribute to an age-related loss of shortening velocity as well as force generation. It also means physiological CSA will not drop as dramatically as anatomical CSA and actually suggests that the loss of specific torque in pinnate muscles, e.g., plantar flexors and knee extensors, is *greater* than that calculated using anatomical CSA as the divisor.

The decrease in fascicle length, and hence in number of sarcomeres in series, will reduce maximum shortening velocity. However, the observed impact on shortening velocity with aging exceeds that which could be explained by decreasing fascicle length. In one study a 38% decrease in calculated maximum shortening velocity of human gastrocnemius was reduced to a 16% decrease when adjusted for fascicle length, suggesting further age-related factors intrinsic to muscle play a role (Thom et al., 2007)

### Changes in fiber morphology

Light microscopic investigation of whole-muscle slices of male human cadaveric vastus lateralis showed that the decrease in CSA with age was in part due to a loss in number of fibers; a 50% reduction in fiber numbers was seen between mean age 19 and 82 years in this sample which did show a greater than normal loss of muscle bulk. No particular fiber type was lost preferentially. However whilst type I fiber size remained constant with age, there was a significant decrease in type II fiber diameter. With advanced age there was also an accumulation of abnormal fibers; they tended to be small and angular and were found individually or in groups of similar such fibers. There were also some large hypertrophic fibers (Lexell et al., 1983, 1988). As described below these shrunken angular fibers are characteristic of denervated fibers.

There is further evidence supporting a selective decrease in size of type II fibers (Larsson, 1978; Coggan et al., 1992; Larsson et al., 1997; Cristea et al., 2010) though some also suggest a decrease in diameter of type I fibers (D’Antona et al., 2003). Another histological study suggested that whilst type II fiber size reduce by c. 57%, type I fibers shrunk by 25% between the third and ninth decades (Andersen, 2003). There is evidence that there may be sexual dimorphism in this phenomenon; it has been observed that type I fiber size remained constant with aging in both sexes with a significant decrease in type II fiber size observed only in males (Yu et al., 2007). Some small biopsy-based studies suggest a preferential loss of type II fibers and subsequent increase in proportion of type I but these should be interpreted with caution given the lack of fiber type homogeneity across muscle seen in old age (Larsson et al., 1997). Discrepancies between studies observing the impact of age on fiber size and type are discussed below.

### Changes in myonuclei and satellite cells

A widely accepted paradigm in understanding myonuclear function proposes the constancy of the MND; with hypertrophy new myonuclei arise from satellite cell turnover and with hypoplasia myonuclei apoptose (Hall and Ralston, 1989). This has been called into question by observations of increased density of nuclei and hence reduced MND in disuse atrophy (Gundersen and Bruusgaard, 2008) and old age (Kadi et al., 2004) which has lead to the suggestion of decreased nuclear efficiency with age. However the most recent developments in assessing the spatial arrangement of myonuclei have demonstrated little or no change in average MND size with aging. Instead there is an apparent increase in the *variability* of size of MND and a clustering of myonuclei within grooves on the periphery of the fiber which may itself have implications in the efficiency of the myonuclear control of muscle protein synthesis (MPS; Cristea et al., 2010).

The number of satellite cells per muscle fiber has been shown to decrease by 24% in women and 37% in men when those aged 20–32 years were compared to those 70–83 years. Due to the increase in myonuclear density in this study there was an even larger decrease in the ratio of satellite cells to myonuclei. It is proposed this may lead to a loss of regenerative function (Kadi et al., 2004).

### Changes in fiber biochemistry

In young humans most muscle fibers express a single MyHC isoform though different isoforms do coexist in some fibers, with type I/IIA and IIA/IIx being recognized and having contractile properties intermediate between classical fiber types (Larsson et al., 1997). In aged muscle there is a greater tendency for fibers to express a mixed pattern of MyHC isoforms. Within aged vastus lateralis, 20–28% of fibers were seen to express both type I and type IIA and 22–33% expressed both type IIA and type IIx. Some individual fibers expressing all three isoforms have been identified in biopsies of aged biceps brachii and vastus lateralis, a phenomenon not seen in young muscle (Klitgaard et al., 1990; Andersen, 2003). Other unusual hybrids seen in old age include type I/IIx and expression of neonatal myosin was unexpectedly observed (D’Antona et al., 2003).

A shift toward slow myosin isoform expression is seen in some studies (Gelfi et al., 2006) but not others (Marx et al., 2002; D’Antona et al., 2003). Some of the discrepancy between studies exploring the impact of age on fiber type and myosin isoform expression has been explained by D’Antona et al. They demonstrated dramatically different biochemical and morphological features when comparing muscle from elderly and elderly immobile patients. Immobility accelerated the observed reduction in fiber CSA, in myosin concentration and in specific tension. However, the elderly immobile had a shift toward *fast* isoform expression. MyHC 2X became the most plentiful isoform and fibers co-expressing IIA/X outnumbered those expressing type I. Accompanying this was the paradoxical *increase* in maximum shortening velocity of isolated skinned fibers from the old immobile even when compared to young controls. These data are consistent with observations made in “unloaded” muscle, e.g., during spaceflight or limb immobilization, of muscle wasting accompanied by a shift toward a fast twitch phenotype. Thus it is proposed that the actual expression pattern of myosin isoforms in the elderly is complex because it depends upon conflicting influences of aging and reduced activity tending to shift toward slow and fast isoforms, respectively (D’Antona et al., 2003).

Muscle architecture changes with age. Fibers become shorter as well as thinner and few in number. There is an accumulation of abnormal shrunken angular fibers both individually and in groups and there is clustering of fiber types. These changes suggest denervation and incomplete reinnervation. Type II fibers are more vulnerable to hypoplasia than type I. Fiber classification by MyHC isoform becomes less relevant as fiber increasingly expressed mixed characteristics. Aging and activity levels influence the pattern of MyHC isoform expression. Whist the average MND size changes little with age, its variability increases with age due to clustering of myonuclei.

## Underlying Mechanisms of Sarcopenia

### Genetic determinants of sacropenia

Sarcopenia is determined by peak muscle mass and the subsequent rate of loss. The degree to which human skeletal muscle phenotypes are heritable has been widely explored. In young people, muscle size and strength are strongly heritable characteristics with *h*^2^ of c. 90% in males in their 20s (Huygens et al., 2004) and in girls and boys aged 10–14 years (Loos et al., 1997). In older people a much smaller proportion of variance in muscle mass and strength is attributable to genetics, with *h*^2^ for leg lean mass as low as 5% and hand grip strength 22%. Environmental factors explain most of the variability (Carmelli and Reed, 2000; Prior et al., 2007). It has been shown in a longitudinal study of male twins, baseline age 63 years, that the proportion of variance in hand grip strength due to genetics decreases from 35 to 22% over a 10 year period (Carmelli and Reed, 2000).

It is therefore unsurprising that the study the molecular genetic basis of sarcopenia remains an area of active research with results of studies identifying “sarcopenia genes” being at best tentative and occasionally inconsistent. The subject has recently been comprehensively reviewed (Garatachea and Lucia, 2011; Tan et al., 2012), with only five genes being identified as having contributed to variation in skeletal muscle mass or strength, in two or more studies. These are angiotensin 1 converting enzyme 1 (ACE), alpha actinin 3 (ACTN3), myostatin (MSTN), ciliary neurotrophic factor (CNTF), and vitamin D receptor (VDR). Linkage and association findings suggest insulin-like growth factor 1 (IGF-1), androgen receptor (AR), and interleukin 6 (IL-6) genes may also contribute to variation in muscle phenotypes.

It cannot be assumed that these genes will subsequently be shown to play a direct role in the pathogenesis of sarcopenia. MSTN attracted early attention as a sarcopenia gene. It’s product MSTN was suggested to contribute to sarcopenia after it was shown that MSTN deficient mice did not experience the same age-related muscle atrophy as wild-type controls (Siriett et al., 2006). Anti-MSTN antibodies could even increase muscle mass and strength in an adult mouse model (Whittemore et al., 2003). Further, MSTN mRNA and protein expression levels appeared to increase in older men (Leger et al., 2008). However the hypothesis that increasing MSTN expression significantly contributes to sarcopenia has subsequently been refuted by a larger study comparing young and frail old men that showed no association between serum MSTN levels and knee extensor strength (*r*^2^ = 0.0001, *p* = 0.97) or quadriceps CSA (*r*^2^ = 0.0121, *p* = 0.48; Ratkevicius et al., 2011).

No single “unfavorable” genotype, associated with accelerated sarcopenia, is yet supported by solid data (Garatachea and Lucia, 2011). The strongest candidate to date is a Lys(K)153Arg(R) polymorphism in exon 2 of the MSTN gene. Studying large groups with this polymorphism has been difficult as the frequency of the mutant R allele is <5% in Caucasians and <20% in Africans. However there is evidence that the KR and RR phenotypes confer weakness on aged women (Seibert et al., 2001; Gonzalez-Freire et al., 2010).

### Alterations in protein synthesis

#### Protein balance

Muscles exist in a dynamic equilibrium with constant MPS and breakdown (MPB). To achieve proteostasis the two are closely matched, though diurnal fluctuations reflect normal intermittent feeding. In the fasted state and at rest, about 0.05% of the myofibrilar mass of leg muscles is synthesized each hour and this rate has not been shown to differ between young and old men. However in young men this rate more than doubles upon either feeding or exercise but in old men these responses are blunted (Cuthbertson et al., 2005; Kumar et al., 2009). As the age-related loss of muscle mass must reflect a period when average MPB exceeds MPS, a blunting of the anabolic response to these stimuli may be an important contributor to sarcopenia.

Further, MPB is inhibited in the postprandial period, mainly due to the availability of insulin. The ability of insulin to suppress leg muscle proteolysis has been shown to be diminished in older people. Thus there may a compromise in both the downregulation of MPB *and* the upregulation in MPS in response to feeding (Wilkes et al., 2009).

#### Changes to the proteome

Whilst loss of muscle mass requires protein breakdown to exceed synthesis, a loss of muscle quality of specific tension suggests a change in the profile of protein synthesis, i.e., the proteome. This has been explored using two-dimensional difference gel electrophoresis as a quantitative differential analysis of protein expression to comparing young and aged human skeletal muscle. Numerous proteins show differential expression dependent upon age. Most are either components of the contractile system or enzymes involved in cellular metabolism (Gelfi et al., 2006).

Myosin heavy chain isoform expression changed dramatically with age. With aging there was an increase in the proportion of MyHC 1 from 48 to 68%; a decrease in the proportion of MyHC 2A from 39 to 31% and a near total loss of MyHC 2X, from 13 to 1%. Myosin light chain (MLC) expression patterns were also different, with a shift away from expression of a fast isoform Q14843 toward P10916, associated with slower twitch velocity. MLC phosphorylation was also lower in the elderly. *In vitro* studies suggest MLC phosphorylation increases calcium sensitivity (Sweeney et al., 1993). Further, troponin T and tropomyosis α-chain isoform, both known to confer calcium sensitivity, are downregulated with advancing age. Reduced calcium sensitivity and reduced calcium release due to dihydropyridine-ryanodine receptor uncoupling will result in excitation-contraction uncoupling (Delbono et al., 1995).

Most of those enzymes that are downregulated with aging participate in anaerobic metabolism. These include creatine kinase as well as several enzymes of the glycolysis pathway including glycogen phosphorylase, glyceraldehydes-3-phosphate dehydrogenase, triosephosphate isomerizes, and δ-enolase. Conversely several enzymes with roles in aerobic metabolism were upregulated in the elderly. These included ATP synthase δ-chain, dihydrolipoamide dehydrogenase, three isoforms of aconitase, malate dehydrogenase, and ubiquinol-cytochrome C reductase complex.

Together these proteomic observations back the *in vivo* observation of older muscles loosing strength and slowing in contraction velocity and are also consistent with the observed decreases in fatigability. The observation of decreased MyHC IIA and IIx expression has been described in some (Larsson et al., 1997) but not all studies (Marx et al., 2002; D’Antona et al., 2003) is discussed above.

### Changes in the hormone and cytokine milieu

There are marked endocrine changes associated with advancing age (Lamberts et al., 1997). At menopause, there is an abrupt decrease in ovarian estrogen production. As described above, there is little evidence that this has a dramatic effect upon maintenance of muscle mass or strength.

A much more gradual but nonetheless significant decrease in testosterone and adrenal androgens is seen in males; this has been referred to as the *andropause*. Given the potent anabolic effects of testosterone (Urban et al., 1995), its decreased production may contribute to sarcopenia in males who do loose muscle mass and strength faster than women as described above. Bioavailable testosterone levels have been shown to positively correlate with peak knee extensor strength in older males (Haren et al., 2008).

Dehydoepiandrosterone (DHEA) is the most plentiful circulating steroid hormones and its levels also decrease with advancing age (Ganong, 2005). Most is of adrenal origin; as DHEA itself is weakly androgenic and its metabolites include active androgens, there has been much interest in the potential significance of its decline in later years and the role of therapeutic administration (Casson et al., 1998; Flynn et al., 1999; Percheron et al., 2003). As yet there is little evidence to supporting the hypothesis that DHEA decreases directly contribute to sarcopenia.

Activity in the pituitary growth hormone (GH)/hepatic IGF-1 axis also diminishes with age and this has been termed the *somatopause* (Lamberts et al., 1997). The ability of subcutaneous GH administration to increase lean mass and reduce fat mass in older men suggests that its decrease with aging may contribute to sarcopenia and the body composition changes seen in old age (Rudman et al., 1990).

High parathyroid hormone levels (≥4 pmol/L) approximately double the risk of sarcopenia (Visser et al., 2003).

Chronic inflammation and subsequent cytokine production has received attention as a potential contributor to sarcopenia. There is an age-related increase in circulating levels of the mildly catabolic IL-6 (Roubenoff et al., 1998; Pedersen et al., 2003). Il-6 levels have been shown to negatively correlate with quadriceps strength in healthy older people (Yende et al., 2006) and muscle function in elderly hospitalized patients (Bautmans et al., 2005). Some (Pedersen et al., 2003) but not all (Roubenoff et al., 1998) studies observed age-related increases in the more markedly catabolic, pro-apoptotic cytokine tumor necrosis factor (TNF)-α. Consistent with an age-related increase in chronic low grade inflammation within muscles and this playing a part in the development of sarcopenia is the observation of increasing expression of genes such as compliment C1QA, galectin-1, and FOXO3A which are involved in inflammatory signaling and apoptosis (Giresi et al., 2005).

IL-6 has been shown to downregulate IGF-1 levels both *in vivo* and *in vitro* (De Benedetti et al., 1997; Lelbach et al., 2001), suggesting a biological link between these factors. Further, the impact of elevated IL-6 and low IGF-1 (both individually and combined) on mobility, independence and survival among older women has been examined. Women in the highest quartile of IL-6 or the lowest quartile of IGF-1 had significantly compromised mobility when compared to those with neither risk factor and women with both risk factors had more disability and higher mortality (Cappola et al., 2003), suggesting a compounding impact of dysregulation in immunological and endocrine systems.

It is widely acknowledged that changes in the endocrine milieu, especially decreased androgens and GH/IGF-1 are contributing factor to sarcopenia (Doherty, 2003; Volpi et al., 2004; Narici and Maffulli, 2010). Increased catabolic cytokine signaling may also contribute though it is likely that this plays a more major role in those elderly patients with accelerated muscle loss associated with chronic inflammatory conditions such as chronic obstructive airway disease or rheumatoid arthritis.

### Loss of innervations

It has long been recognized that the pattern of histological changes seen in muscle, described above, suggested that denervation significantly contributed to wasting. The terms “disseminated neurogenic atrophy” and “grouped denervation atrophy” were used to describe the progressive accumulation and clustering of small, angular fibers with denervated appearance (Tomlinson et al., 1969). Alongside this there is a progressive loss of α-motoneurons. The total number of limb motoneurons in the human lumbosacral region of the human spinal cord was found to average at 57–60,000 before 60 years dropping to 45,000 in octogenarians and 40,000 in non-agenarians (Tomlinson and Irving, 1977).

Electrophysiological studies have confirmed a decrease in the number of motor units with aging (Brown, 1972). Alongside a reduction in number of motor units is some increase in size of motor units (Doherty et al., 1993), suggesting some reinnervation effort. Studies suggest a compromised potential for terminal sprouting and reinnervation of muscle fibers, at least in aged rodentia (Einsiedel and Luff, 1992) Further evidence supporting rounds of denervation and reinnervation is based on the observation that the spatial distribution of fibers in each motor unit in rat muscle becomes more clustered with advancing age (Larsson, 1995). In young humans, fiber types appear randomly distributed across the muscle but they become increasingly grouped or clustered together in age. This may reflect a similar process of denervation and reinnervation (Nygaard and Sanchez, 1982; Lexell and Downham, 1991; Andersen, 2003).

Fewer, larger motor units have been observed in muscles in human upper and lower limbs, especially in smaller distal muscles though it may be a less pronounced phenomenon in more proximal muscles such as biceps brachii (Campbell et al., 1973; Galea, 1996).

It is therefore proposed that apoptosis of motoneurons with subsequent incomplete reinnervation of fibers by surviving neurons is a major contributor to the loss of strength and muscle mass with age (Luff, 1998).

### Nutrition and activity levels

Despite the observed increases in body fat and obesity seen in old age, there is a decrease in food intake across the adult lifespan. The reasons for this anorexia of old age are complex and include visceral, hormonal, neurological, pharmacological, and psychological factors (Morley, 1997). In a survey of 60 years+ people in a developed country, over 50% had a usual intake of less than 1.0 g of high quality protein per kg body weight per day (Sayhoun, 1992). In another survey, 30% had less than 0.8 g/kg/day and 15% less than 0.6 g/kg/day (Roubenoff and Hughes, 2000). It has been suggested that the usual recommended daily amount of protein, 0.8 g/kg/day, is an underestimate and in old age 1.25 g could be considered an appropriate value (Evans and Cyr-Campbell, 1997).

An intake of 0.45 g/kg/day has shown to lead to dramatic and rapid loss of lean tissue and muscle function in older ladies (Castaneda et al., 1995). Dietary supplementation with oral amino acids, providing an additional 0.25 g/kg/day of protein has been shown to increase lean mass in sarcopenic patients (Solerte et al., 2008).

Caloric intake also decreases and this can contribute to sarcopenia as a negative energy balance will result in a negative nitrogen balance regardless of actual nitrogen intake (Roberts, 1995).

Further, vitamin D deficiency (serum 25-OHD ≤ 25 nmol/L) has been shown to more than double the risk of sarcopenia (Visser et al., 2003).

Since sarcopenia was first conceived as a concept there has been the realization that decreasing physical activity played a role in its development and held potential as an intervention (Rosenberg, 1989; Evans and Campbell, 1993; Evans and Cyr-Campbell, 1997). The role of exercise as an intervention is discussed below.

### Compromised vascularization

Capillary density (/mm) has been seen to decrease with age in sedentary individuals (Coggan et al., 1992) but not in masters athletes (Coggan et al., 1990) but these findings are not consistent. Other studies show that a decrease in capillaries per fiber seen with aging is largely (Frontera et al., 1990) or entirely (Andersen, 2003) due to fibers being smaller and the resulting capillary density is unchanged. Despite the apparent preservation of capillary density there is a reduction in the increase in leg bulk flow on exercise (Proctor et al., 1998).

This may be due to compromised vasomotor responsiveness in age rather than reduced capillary numbers. There is evidence of a lower sensitivity of arteriolar tone to normal vasodilatory stimuli; endothelium dependent and nitric-oxide mediated; in arterioles from the mainly slow twitch, oxidative rat soleus. There may be heterogeneity between muscle types as this phenomenon was not observed in the fast twitch glycolytic gastrocnemius (Muller-Delp et al., 2002).

Compromised vascular responsiveness would expose aged muscles to potential hypoxia, free radical stress, and could compromise nutrient delivery.

### Increased oxidative stress

With aging a reduction in the cellular anti-oxidant buffering mechanisms and an increase in the generation of free radicals due to dysfunction in the mitochondrial respiratory chain result in an increase in the oxidative stress to which the cell is exposed (Barreiro et al., 2006). This will result in damage to muscle components including DNA, myofibrilar, and mitochondrial proteins, the neuromuscular junction, and those elements of the sarcoplasmic reticulum which are responsible for the Ca^2+^ release that initiates contraction. It may contribute to α-motoneuron atrophy and the reduced number and function of satellite cells seen in old age. Levels of serum protein carbonyls, a measure of oxidative stress, are a strong predictor of weak hand grip strength in elderly women (Howard et al., 2007).

It has been proposed that damage to mitochondrial DNA may play a significant part in muscle aging. This tightly packed DNA resides close to the main generator of reactive oxygen species and is therefore at risk of accumulating genetic lesions. These genetic insults may lead to further mitochondrial dysfunction and a vicious-cycle of increasing oxidative stress (Hiona and Leeuwenburgh, 2008).

There is an important heritable element in aging skeletal muscle phenotypes but data published to date on individual genes tends to be tentative and controversial. These are likely polygenic traits and thus not reducible to specific polymorphisms. With increasing age the muscle phenotype is increasingly shaped by environment.

Older muscles demonstrate a blunted anabolic response to feeding and exercise. Enzymes involved in anaerobic metabolism are expressed less in old muscle whilst the aerobic pathways are preserved or enhanced. In healthy active aging, myosin isoform expression shifts away from fast type 2A and 2X and toward slower type I.

The production of anabolic hormones such as testosterone and IGF-1 drop in old age. Levels of the catabolic cytokines IL-6 increase.

Spinal α-motoneurons are lost with age resulting in fewer motor units. There is some increase in size of motor units but there is still histological evidence of denervation. Older people eat less protein and are less active than young people. Blood vessels are less able to respond to demand for changing blood flow. Oxidative stress increases in aged muscle and free radical damage ensues. The mechanisms that contribute to sarcopenia are complex, overlapping, and interdependent.

## Interventions in Sarcopenia

A wealth of literature supports the efficacy of resistive exercise for combating sarcopenia (Meltzer, 1994). For instance, 12 months resistive training in septuagenarian males was found to increase calf muscle volume by 12% and torque by 20% (Morse et al., 2005). Instead, older women (aged 80 years+) display a blunted anabolic response, as no increase in muscle size was found after 12 weeks of resistive training (Raue et al., 2009). Nevertheless, from a systematic review of 120 trials with 6700 older participants, it has been found that the gains in muscle mass and strength afforded by resistive training are associated with a small but significant improvement in physical performance (e.g., gait and ability to raise from a chair; Liu and Latham, 2009). It is noteworthy that lower intensity mechanical loading such as aerobic exercise, despite being considerably less effective for inducing muscle hypertrophy, has been found to promote protein synthesis and expression of growth-related genes and inhibit the expression of muscle breakdown-related genes (Harber et al., 2009). Also, exciting new research findings suggest that aerobic exercise (running) activity sustained for decades (as that performed by master athletes) affords protection against the age-related loss of motor unit number. These findings show that the number of motor units in the tibialis anterior muscle of master runners was similar to that of young runners and significantly higher than that of age-matched older inactive controls. These neuro-protective benefits of running seem restricted to the exercised muscles of the lower limbs since they were absent in the upper limbs (Power et al., 2012). Furthermore, since aerobic exercise training is a potent and effective intervention for the prevention and treatment of insulin resistance, it is likely also to provide protection against the blunting of insulin inhibition of proteolysis in old age, contributing therefore to prevent age-related sarcopenia (Wilkes et al., 2009).

The associated concepts of sarcopenia and dynapenia remain exciting fields of research. We need to refine prediction models for disability, institutionalization and mortality, and translate these into clinical practice. Through exercise based studies we must explore the degree of reversibility of the sarcopenic and dynapenic states. Successful interventions may allow us to infer causality between loss of muscle and strength and the associated health and survival impact. Evidence supporting the use of nutritional, pharmacological, hormonal, and anti-oxidant therapies may revolutionize the approach to treating sarcopenia.

## Conclusion

With old age skeletal muscles get weaker and they get smaller but they get weaker much faster than they get smaller. The force generated per unit CSA decreases as muscle quality changes with age. Numerous underlying mechanisms contribute to sarcopenia. Evidence supports a role for resistive exercise training in combating sarcopenia. Nutritional and pharmacological interventions remain areas of active research.

## Conflict of Interest Statement

The authors declare that the research was conducted in the absence of any commercial or financial relationships that could be construed as a potential conflict of interest.

## References

[B1] Al Snih S. Markides K. S. Ottenbacher K. J. Raji M. A. (2004). Hand Grip strength and incident ADL disability in elderly Mexican Americans over a seven-year period. Aging Clin. Exp. Res. 16, 481–486 15739601 10.1007/BF03327406

[B2] Aloia J. F. Mcgowan D. M. Vaswani A. N. Ross P. Cohn S. H. (1991). Relationship of menopause to skeletal and muscle mass. Am. J. Clin. Nutr. 53, 1378–1383 2035465 10.1093/ajcn/53.6.1378

[B3] Andersen J. L. (2003). Muscle fibre type adaptation in the elderly human muscle. Scand. J. Med. Sci. Sports 13, 40–47 10.1034/j.1600-0838.2003.00299.x 12535316

[B4] Avin K. G. Law L. A. (2011). Age-related differences in muscle fatigue vary by contraction type: a meta-analysis. Phys. Ther. 91, 1153–1165 10.2522/ptj.20100333 21616932 PMC3145894

[B5] Bajekal M. Wheeler L. Dix D. (2006) Estimating residents and staff in communal establishments from the 2001 census. Health Stat. Q. 31, 42–50 16972695

[B6] Barreiro E. Coronell C. Lavina B. Ramirez-Sarmiento A. Orozco-Levi M. Gea J. (2006). Aging, sex differences, and oxidative stress in human respiratory and limb muscles. Free Radic. Biol. Med. 41, 797–809 10.1016/j.freeradbiomed.2006.05.027 16895800

[B7] Baumgartner R. N. Koehler K. M. Gallagher D. Romero L. Heymsfield S. B. Ross R. R. Garry P. J. Lindeman R. D. (1998). Epidemiology of sarcopenia among the elderly in New Mexico. Am. J. Epidemiol. 147, 755–763 10.1093/oxfordjournals.aje.a009520 9554417

[B8] Baumgartner R. N. Stauber P. M. Mchugh D. Koehler K. M. Garry P. J. (1995). Cross-sectional age differences in body composition in persons 60+ years of age. J. Gerontol. A Biol. Sci. Med. Sci. 50, M307–M316 10.1093/gerona/50A.6.M307 7583802

[B9] Bautmans I. Njemini R. Lambert M. Demanet C. Mets T. (2005). Circulating acute phase mediators and skeletal muscle performance in hospitalized geriatric patients. J. Gerontol. A Biol. Sci. Med. Sci. 60, 361–367 10.1093/gerona/60.3.361 15860475

[B10] Bigland-Ritchie B. Woods J. J. (1984). Changes in muscle contractile properties and neural control during human muscular fatigue. Muscle Nerve 7, 691–699 10.1002/mus.880070902 6100456

[B11] Borkan G. A. Hults D. E. Gerzof S. G. Robbins A. H. Silbert C. K. (1983). Age changes in body composition revealed by computed tomography. J. Gerontol. 38, 673–677 6630900 10.1093/geronj/38.6.673

[B12] Brown W. F. (1972). A method for estimating the number of motor units in thenar muscles and the changes in motor unit count with ageing. J. Neurol. Neurosurg. Psychiatr. 35, 845–852 10.1136/jnnp.35.6.845 4647858 PMC494191

[B13] Campbell M. J. Mccomas A. J. Petito F. (1973). Physiological changes in ageing muscles. J. Neurol. Neurosurg. Psychiatr. 36, 174–182 10.1136/jnnp.36.2.174 4708452 PMC1083551

[B14] Cappola A. R. Xue Q. L. Ferrucci L. Guralnik J. M. Volpato S. Fried L. P. (2003). Insulin-like growth factor I and interleukin-6 contribute synergistically to disability and mortality in older women. J. Clin. Endocrinol. Metab. 88, 2019–2025 10.1210/jc.2003-030398 12727948

[B15] Carmelli D. Reed T. (2000). Stability and change in genetic and environmental influences on hand-grip strength in older male twins. J. Appl. Physiol. 89, 1879–1883 11053339 10.1152/jappl.2000.89.5.1879

[B16] Casson P. R. Santoro N. Elkind-Hirsch K. Carson S. A. Hornsby P. J. Abraham G. Buster J. E. (1998). Postmenopausal dehydroepiandrosterone administration increases free insulin-like growth factor-I and decreases high-density lipoprotein: a six-month trial. Fertil. Steril. 70, 107–110 10.1016/S0015-0282(98)00121-6 9660430

[B17] Castaneda C. Charnley J. M. Evans W. J. Crim M. C. (1995). Elderly women accommodate to a low-protein diet with losses of body cell mass, muscle function, and immune response. Am. J. Clin. Nutr. 62, 30–39 7598064 10.1093/ajcn/62.1.30

[B18] Cawthon P. M. Fox K. M. Gandra S. R. Delmonico M. J. Chiou C. F. Anthony M. S. Sewall A. Goodpaster B. Satterfield S. Cummings S. R. Harris T. B. (2009). Do muscle mass, muscle density, strength, and physical function similarly influence risk of hospitalization in older adults? J. Am. Geriatr. Soc. 57, 1411–1419 10.1111/j.1532-5415.2009.02366.x 19682143 PMC3269169

[B19] Chien M. Y. Huang T. Y. Wu Y. T. (2008). Prevalence of sarcopenia estimated using a bioelectrical impedance analysis prediction equation in community-dwelling elderly people in Taiwan. J. Am. Geriatr. Soc. 56, 1710–1715 10.1111/j.1532-5415.2008.01854.x 18691288

[B20] Clark B. C. Manini T. M. (2008). Sarcopenia =/= dynapenia. J. Gerontol. A Biol. Sci. Med. Sci. 63, 829–834 10.1093/gerona/63.8.829 18772470

[B21] Clark B. C. Manini T. M. (2010). Functional consequences of sarcopenia and dynapenia in the elderly. Curr. Opin. Clin. Nutr. Metab. Care 13, 271–276 10.1097/MCO.0b013e328337819e 20154609 PMC2895460

[B22] Clement F. J. (1974). Longitudinal and cross-sectional assessments of age changes in physical strength as related to sex, social class, and mental ability. J. Gerontol. 29, 423–429 4833758 10.1093/geronj/29.4.423

[B23] Coggan A. R. Spina R. J. King D. S. Rogers M. A. Brown M. Nemeth P. M. Holloszy J. O. (1992). Histochemical and enzymatic comparison of the gastrocnemius muscle of young and elderly men and women. J. Gerontol. 47, B71–B76 1573181 10.1093/geronj/47.3.b71

[B24] Coggan A. R. Spina R. J. Rogers M. A. King D. S. Brown M. Nemeth P. M. Holloszy J. O. (1990). Histochemical and enzymatic characteristics of skeletal muscle in master athletes. J. Appl. Physiol. 68, 1896–1901 10.1063/1.346579 2361892

[B25] Cohn S. H. Vartsky D. Yasumura S. Sawitsky A. Zanzi I. Vaswani A. Ellis K. J. (1980). Compartmental body composition based on total-body nitrogen, potassium, and calcium. Am. J. Physiol. 239, E524–E530 7446727 10.1152/ajpendo.1980.239.6.E524

[B26] Cristea A. Qaisar R. Edlund P. K. Lindblad J. Bengtsson E. Larsson L. (2010). Effects of aging and gender on the spatial organization of nuclei in single human skeletal muscle cells. Aging Cell 9, 685–697 10.1111/j.1474-9726.2010.00594.x 20633000

[B27] Cruz-Jentoft A. J. Baeyens J. P. Bauer J. M. Boirie Y. Cederholm T. Landi F. Martin F. C. Michel J. P. Rolland Y. Schneider S. M. Topinkova E. Vandewoude M. Zamboni M. (2010). Sarcopenia: European consensus on definition and diagnosis: Report of the European Working Group on Sarcopenia in Older People. Age Ageing 39, 412–423 10.1093/ageing/afq034 20392703 PMC2886201

[B28] Cuthbertson D. Smith K. Babraj J. Leese G. Waddell T. Atherton P. Wackerhage H. Taylor P. M. Rennie M. J. (2005). Anabolic signaling deficits underlie amino acid resistance of wasting, aging muscle. FASEB J. 19, 422–424 15596483 10.1096/fj.04-2640fje

[B29] D’Antona G. Pellegrino M. A. Adami R. Rossi R. Carlizzi C. N. Canepari M. Saltin B. Bottinelli R. (2003). The effect of ageing and immobilization on structure and function of human skeletal muscle fibres. J. Physiol. (Lond.) 552, 499–511 10.1113/jphysiol.2003.046276 14561832 PMC2343394

[B30] Davies Re C. N. Gergely J. (2012). Encyclopedia Britannica Online Academic Edition. Available at: http://www.britannica.com/EBchecked/topic/524076/sarcomere

[B31] De Benedetti F. Alonzi T. Moretta A. Lazzaro D. Costa P. Poli V. Martini A. Ciliberto G. Fattori E. (1997). Interleukin 6 causes growth impairment in transgenic mice through a decrease in insulin-like growth factor-I. A model for stunted growth in children with chronic inflammation. J. Clin. Invest. 99, 643–650 10.1172/JCI119207 9045866 PMC507846

[B32] Delbono O. O’Rourke K. S. Ettinger W. H. (1995). Excitation-calcium release uncoupling in aged single human skeletal muscle fibers. J. Membr. Biol. 148, 211–222 8747553 10.1007/BF00235039

[B33] Delmonico M. J. Harris T. B. Lee J. S. Visser M. Nevitt M. Kritchevsky S. B. Tylavsky F. A. Newman A. B. (2007). Alternative definitions of sarcopenia, lower extremity performance, and functional impairment with aging in older men and women. J. Am. Geriatr. Soc. 55, 769–774 10.1111/j.1532-5415.2007.01140.x 17493199

[B34] Delmonico M. J. Harris T. B. Visser M. Park S. W. Conroy M. B. Velasquez-Mieyer P. Boudreau R. Manini T. M. Nevitt M. Newman A. B. Goodpaster B. H. (2009). Longitudinal study of muscle strength, quality, and adipose tissue infiltration. Am. J. Clin. Nutr. 90, 1579–1585 10.3945/ajcn.2009.28047 19864405 PMC2777469

[B35] Dey D. K. Bosaeus I. Lissner L. Steen B. (2009). Changes in body composition and its relation to muscle strength in 75-year-old men and women: a 5-year prospective follow-up study of the NORA cohort in Goteborg, Sweden. Nutrition 25, 613–619 10.1016/j.nut.2008.11.023 19211225

[B36] Doherty T. J. (2003). Invited review: aging and sarcopenia. J. Appl. Physiol. 95, 1717–1727 12970377 10.1152/japplphysiol.00347.2003

[B37] Doherty T. J. Vandervoort A. A. Taylor A. W. Brown W. F. (1993). Effects of motor unit losses on strength in older men and women. J. Appl. Physiol. 74, 868–874 10.1063/1.354879 8458808

[B38] Douchi T. Yamamoto S. Yoshimitsu N. Andoh T. Matsuo T. Nagata Y. (2002). Relative contribution of aging and menopause to changes in lean and fat mass in segmental regions. Maturitas 42, 301–306 10.1016/S0378-5122(02)00004-X 12191853

[B39] Einsiedel L. J. Luff A. R. (1992). Effect of partial denervation on motor units in the ageing rat medial gastrocnemius. J. Neurol. Sci. 112, 178–184 10.1016/0022-510X(92)90148-E 1469430

[B40] Evans W. J. (1995). What is sarcopenia? J. Gerontol. A Biol. Sci. Med. Sci. 50, 5–8 10.1093/gerona/50A.Special_Issue.5 7493218

[B41] Evans W. J. Campbell W. W. (1993). Sarcopenia and age-related changes in body composition and functional capacity. J. Nutr. 123, 465–468 8429405 10.1093/jn/123.suppl_2.465

[B42] Evans W. J. Cyr-Campbell D. (1997). Nutrition, exercise, and healthy aging. J. Am. Diet. Assoc. 97, 632–638 10.1016/S0002-8223(97)00160-0 9183325

[B43] Fischer M. Birren J. (1946). Standardization, of a test of hand strength. J. Appl. Psychol. 30, 380–387 10.1037/h0059969 20998487

[B44] Flynn M. A. Weaver-Osterholtz D. Sharpe-Timms K. L. Allen S. Krause G. (1999). Dehydroepiandrosterone replacement in aging humans. J. Clin. Endocrinol. Metab. 84, 1527–1533 10.1210/jc.84.5.1527 10323374

[B45] Frey Law L. A. Avin K. G. (2010). Endurance time is joint-specific: a modelling and meta-analysis investigation. Ergonomics 53, 109–129 10.1080/00140130903389068 20069487 PMC2891087

[B46] Frontera W. R. Hughes V. A. Fielding R. A. Fiatarone M. A. Evans W. J. Roubenoff R. (2000). Aging of skeletal muscle: a 12-yr longitudinal study. J. Appl. Physiol. 88, 1321–1326 10749826 10.1152/jappl.2000.88.4.1321

[B47] Frontera W. R. Hughes V. A. Lutz K. J. Evans W. J. (1991). A cross-sectional study of muscle strength and mass in 45- to 78-yr-old men and women. J. Appl. Physiol. 71, 644–650 1938738 10.1152/jappl.1991.71.2.644

[B48] Frontera W. R. Meredith C. N. O’Reilly K. P. Evans W. J. (1990). Strength training and determinants of VO2max in older men. J. Appl. Physiol. 68, 329–333 2312474 10.1152/jappl.1990.68.1.329

[B49] Galea V. (1996). Changes in motor unit estimates with aging. J. Clin. Neurophysiol. 13, 253–260 10.1097/00004691-199605000-00010 8714347

[B50] Gallagher D. Visser M. De Meersman R. E. Sepulveda D. Baumgartner R. N. Pierson R. N. Harris T. Heymsfield S. B. (1997). Appendicular skeletal muscle mass: effects of age, gender, and ethnicity. J. Appl. Physiol. 83, 229–239 9216968 10.1152/jappl.1997.83.1.229

[B51] Ganong W. F. (2005). Review of Medical Physiology. San Francisco: McGraw Hill

[B52] Garatachea N. Lucia A. (2011). Genes and the ageing muscle: a review on genetic association studies. Age (Dordr.). [Epub ahead of print]. 10.1007/s11357-011-9327-0 PMC3543750 22037866

[B53] Gelfi C. Vigano A. Ripamonti M. Pontoglio A. Begum S. Pellegrino M. A. Grassi B. Bottinelli R. Wait R. Cerretelli P. (2006). The human muscle proteome in aging. J. Proteome Res. 5, 1344–1353 10.1021/pr050414x 16739986

[B54] Giresi P. G. Stevenson E. J. Theilhaber J. Koncarevic A. Parkington J. Fielding R. A. Kandarian S. C. (2005). Identification of a molecular signature of sarcopenia. Physiol. Genomics 21, 253–263 10.1152/physiolgenomics.00249.2004 15687482

[B55] Goldspink G. (1985). Malleability of the motor system: a comparative approach. J. Exp. Biol. 115, 375–391 4031777 10.1242/jeb.115.1.375

[B56] Gonzalez-Freire M. Rodriguez-Romo G. Santiago C. Bustamante-Ara N. Yvert T. Gomez-Gallego F. Serra Rexach J. A. Ruiz J. R. Lucia A. (2010). The K153R variant in the myostatin gene and sarcopenia at the end of the human lifespan. Age (Dordr.) 32, 405–409 10.1007/s11357-010-9139-7 20640547 PMC2926851

[B57] Goodpaster B. H. Park S. W. Harris T. B. Kritchevsky S. B. Nevitt M. Schwartz A. V. Simonsick E. M. Tylavsky F. A. Visser M. Newman A. B. (2006). The loss of skeletal muscle strength, mass, and quality in older adults: the health, aging and body composition study. J. Gerontol. A Biol. Sci. Med. Sci. 61, 1059–1064 10.1093/gerona/61.10.1059 17077199

[B58] Gundersen K. Bruusgaard J. C. (2008). Nuclear domains during muscle atrophy: nuclei lost or paradigm lost? J. Physiol. (Lond.) 586, 2675–2681 10.1113/jphysiol.2008.154369 18440990 PMC2536588

[B59] Guyton A. C. (1991). Medical Physiology. Philadelphia: W.B. Saunders

[B60] Hall Z. W. Ralston E. (1989). Nuclear domains in muscle cells. Cell 59, 771–772 10.1016/0092-8674(89)90875-1 2686838

[B61] Harber M. P. Crane J. D. Dickinson J. M. Jemiolo B. Raue U. Trappe T. A. Trappe S. W. (2009). Protein synthesis and the expression of growth-related genes are altered by running in human vastus lateralis and soleus muscles. Am. J. Physiol. Regul. Integr. Comp. Physiol. 296, R708–R714 10.1152/ajpregu.90906.2008 19118097

[B62] Haren M. T. Banks W. A. Perry Iii H. M. Patrick P. Malmstrom T. K. Miller D. K. Morley J. E. (2008). Predictors of serum testosterone and dheas in African-American men. Int. J. Androl. 31, 50–59 18190426 10.1111/j.1365-2605.2007.00757.x PMC2717611

[B63] Hiona A. Leeuwenburgh C. (2008). The role of mitochondrial DNA mutations in aging and sarcopenia: implications for the mitochondrial vicious cycle theory of aging. Exp. Gerontol. 43, 24–33 10.1016/j.exger.2007.10.001 17997255 PMC2225597

[B64] Hook P. Sriramoju V. Larsson L. (2001). Effects of aging on actin sliding speed on myosin from single skeletal muscle cells of mice, rats, and humans. Am. J. Physiol. Cell Physiol. 280, C782–C788 11245594 10.1152/ajpcell.2001.280.4.C782

[B65] Howard C. Ferrucci L. Sun K. Fried L. P. Walston J. Varadhan R. Guralnik J. M. Semba R. D. (2007). Oxidative protein damage is associated with poor grip strength among older women living in the community. J. Appl. Physiol. 103, 17–20 10.1152/japplphysiol.00133.2007 17379753 PMC2646087

[B66] Hughes V. A. Frontera W. R. Roubenoff R. Evans W. J. Singh M. A. (2002). Longitudinal changes in body composition in older men and women: role of body weight change and physical activity. Am. J. Clin. Nutr. 76, 473–481 12145025 10.1093/ajcn/76.2.473

[B67] Huygens W. Thomis M. A. Peeters M. W. Vlietinck R. F. Beunen G. P. (2004). Determinants and upper-limit heritabilities of skeletal muscle mass and strength. Can. J. Appl. Physiol. 29, 186–200 10.1139/h04-014 15064427

[B68] Iannuzzi-Sucich M. Prestwood K. M. Kenny A. M. (2002). Prevalence of sarcopenia and predictors of skeletal muscle mass in healthy, older men and women. J. Gerontol. A Biol. Sci. Med. Sci. 57, M772–M777 10.1093/gerona/57.12.M772 12456735

[B69] Inouye S. K. Studenski S. Tinetti M. E. Kuchel G. A. (2007). Geriatric syndromes: clinical, research, and policy implications of a core geriatric concept. J. Am. Geriatr. Soc. 55, 780–791 10.1111/j.1532-5415.2007.01443.x 17493201 PMC2409147

[B70] Izquierdo M. Ibanez J. Gorostiaga E. Garrues M. Zuniga A. Anton A. Larrion J. L. Hakkinen K. (1999). Maximal strength and power characteristics in isometric and dynamic actions of the upper and lower extremities in middle-aged and older men. Acta Physiol. Scand. 167, 57–68 10.1046/j.1365-201x.1999.00590.x 10519978

[B71] Janssen I. Heymsfield S. B. Ross R. (2002). Low relative skeletal muscle mass (sarcopenia) in older persons is associated with functional impairment and physical disability. J. Am. Geriatr. Soc. 50, 889–896 10.1046/j.1532-5415.2002.50216.x 12028177

[B72] Janssen I. Heymsfield S. B. Wang Z. M. Ross R. (2000). Skeletal muscle mass and distribution in 468 men and women aged 18–88 yr. J. Appl. Physiol. 89, 81–88 10904038 10.1152/jappl.2000.89.1.81

[B73] Kadi F. Charifi N. Denis C. Lexell J. (2004). Satellite cells and myonuclei in young and elderly women and men. Muscle Nerve 29, 120–127 10.1002/mus.10510 14694507

[B74] Kanis J. A. (1994). Assessment of fracture risk and its application to screening for postmenopausal osteoporosis: synopsis of a WHO report. WHO Study Group. Osteoporos. Int. 4, 368–381 10.1007/BF01622200 7696835

[B75] Kehayias J. J. Fiatarone M. A. Zhuang H. Roubenoff R. (1997). Total body potassium and body fat: relevance to aging. Am. J. Clin. Nutr. 66, 904–910 9322566 10.1093/ajcn/66.4.904

[B76] Klass M. Baudry S. Duchateau J. (2005). Aging does not affect voluntary activation of the ankle dorsiflexors during isometric, concentric, and eccentric contractions. J. Appl. Physiol. 99, 31–38 10.1152/japplphysiol.01426.2004 15705734

[B77] Klitgaard H. Zhou M. Schiaffino S. Betto R. Salviati G. Saltin B. (1990). Ageing alters the myosin heavy chain composition of single fibres from human skeletal muscle. Acta Physiol. Scand. 140, 55–62 10.1111/j.1748-1716.1990.tb08975.x 2275405

[B78] Koster A. Ding J. Stenholm S. Caserotti P. Houston D. K. Nicklas B. J. You T. Lee J. S. Visser M. Newman A. B. Schwartz A. V. Cauley J. A. Tylavsky F. A. Goodpaster B. H. Kritchevsky S. B. Harris T. B. (2011). Does the amount of fat mass predict age-related loss of lean mass, muscle strength, and muscle quality in older adults? J. Gerontol. A Biol. Sci. Med. Sci. 66, 888–895 10.1093/gerona/glr070 21572082 PMC3184893

[B79] Kumar V. Selby A. Rankin D. Patel R. Atherton P. Hildebrandt W. Williams J. Smith K. Seynnes O. Hiscock N. Rennie M. J. (2009). Age-related differences in the dose-response relationship of muscle protein synthesis to resistance exercise in young and old men. J. Physiol. (Lond.) 587, 211–217 10.1113/jphysiol.2008.164483 19001042 PMC2670034

[B80] Kyle U. G. Genton L. Hans D. Karsegard L. Slosman D. O. Pichard C. (2001). Age-related differences in fat-free mass, skeletal muscle, body cell mass and fat mass between 18 and 94 years. Eur. J. Clin. Nutr. 55, 663–672 10.1038/sj.ejcn.1601198 11477465

[B81] Lamberts S. W. Van Den Beld A. W. Van Der Lely A. J. (1997). The endocrinology of aging. Science 278, 419–424 10.1126/science.278.5337.419 9334293

[B82] Landi F. Russo A. Liperoti R. Pahor M. Tosato M. Capoluongo E. Bernabei R. Onder G. (2005). Midarm muscle circumference, physical performance and mortality: results from the aging and longevity study in the Sirente geographic area (ilSIRENTE study). Clin. Nutr. 29, 441–447 10.1016/j.clnu.2009.12.006 20116909

[B83] Larsson L. (1978). Morphological and functional characteristics of the ageing skeletal muscle in man. A cross-sectional study. Acta Physiol. Scand. Suppl. 457, 1–36 10.1111/j.1748-1716.1978.tb06041.x 281113

[B84] Larsson L. (1995). Motor units: remodeling in aged animals. J. Gerontol. A Biol. Sci. Med. Sci. 50, 91–95 7493226 10.1093/gerona/50a.special_issue.91

[B85] Larsson L. Karlsson J. (1978). Isometric and dynamic endurance as a function of age and skeletal muscle characteristics. Acta Physiol. Scand. 104, 129–136 10.1111/j.1748-1716.1978.tb06259.x 152565

[B86] Larsson L. Li X. Frontera W. R. (1997). Effects Of aging on shortening velocity and myosin isoform composition in single human skeletal muscle cells. Am. J. Physiol. 272, C638–C649 9124308 10.1152/ajpcell.1997.272.2.C638

[B87] Laukkanen P. Heikkinen E. Kauppinen M. (1995). Muscle strength and mobility as predictors of survival in 75–84-year-old people. Age Ageing 24, 468–473 10.1093/ageing/24.6.468 8588534

[B88] Leger B. Derave W. De Bock K. Hespel P. Russell A. P. (2008). Human sarcopenia reveals an increase in SOCS-3 and myostatin and a reduced efficiency of Akt phosphorylation. Rejuvenation Res. 11, 163–175b 10.1089/rej.2007.0588 18240972

[B89] Lelbach A. Scharf J. G. Ramadori G. (2001). Regulation of insulin-like growth factor-I and of insulin-like growth factor binding protein-1, -3 and -4 in cocultures of rat hepatocytes and Kupffer cells by interleukin-6. J. Hepatol. 35, 558–567 10.1016/S0168-8278(01)00170-2 11690700

[B90] Lexell J. Downham D. Y. (1991). The occurrence of fibre-type grouping in healthy human muscle: a quantitative study of cross-sections of whole vastus lateralis from men between 15 and 83 years. Acta Neuropathol. 81, 377–381 10.1007/BF00293457 2028741

[B91] Lexell J. Henriksson-Larsen K. Winblad B. Sjostrom M. (1983). Distribution of different fiber types in human skeletal muscles: effects of aging studied in whole muscle cross sections. Muscle Nerve 6, 588–595 10.1002/mus.880060809 6646161

[B92] Lexell J. Taylor C. C. Sjostrom M. (1988). What is the cause of the ageing atrophy? Total number, size and proportion of different fiber types studied in whole vastus lateralis muscle from 15- to 83-year-old men. J. Neurol. Sci. 84, 275–294 10.1016/0022-510X(88)90132-3 3379447

[B93] Lieber R. L. Friden J. (2001). Clinical significance of skeletal muscle architecture. Clin. Orthop. Relat. Res. 140–151 10.1097/00003086-200102000-00016 11210948

[B94] Ling C. H. Taekema D. De Craen A. J. Gussekloo J. Westendorp R. G. Maier A. B. (2010). Handgrip strength and mortality in the oldest old population: the Leiden 85-plus study. CMAJ 182, 429–435 10.1503/cmaj.091278 20142372 PMC2842834

[B95] Liu C. J. Latham N. K. (2009). Progressive resistance strength training for improving physical function in older adults. Cochrane Database Syst. Rev. 3, CD002759 19588334 10.1002/14651858.CD002759.pub2 PMC4324332

[B96] Loos R. Thomis M. Maes H. H. Beunen G. Claessens A. L. Derom C. Legius E. Derom R. Vlietinck R. (1997). Gender-specific regional changes in genetic structure of muscularity in early adolescence. J. Appl. Physiol. 82, 1802–1810 9173944 10.1152/jappl.1997.82.6.1802

[B97] Luff A. R. (1998). Age-associated changes in the innervation of muscle fibers and changes in the mechanical properties of motor units. Ann. N. Y. Acad. Sci. 854, 92–101 10.1111/j.1749-6632.1998.tb09895.x 9928423

[B98] Macaluso A. De Vito G. (2004). Muscle strength, power and adaptations to resistance training in older people. Eur. J. Appl. Physiol. 91, 450–472 10.1007/s00421-003-0991-3 14639481

[B99] Marx J. O. Kraemer W. J. Nindl B. C. Larsson L. (2002). Effects Of aging on human skeletal muscle myosin heavy-chain mRNA content and protein isoform expression. J. Gerontol. A Biol. Sci. Med. Sci. 57, B232–B238 10.1093/gerona/57.6.B232 12023259

[B100] Meltzer D. E. (1994). Age dependence of Olympic weightlifting ability. Med. Sci. Sports Exerc. 26, 1053–1067 7968424

[B101] Metter E. J. Talbot L. A. Schrager M. Conwit R. (2002). Skeletal muscle strength as a predictor of all-cause mortality in healthy men. J. Gerontol. A Biol. Sci. Med. Sci. 57, B359–B365 10.1093/gerona/57.10.B359 12242311

[B102] Moore D. H. II. (1975). A study of age group track and field records to relate age and running speed. Nature 253, 264–265 10.1038/253525b0 1113841

[B103] Morley J. E. (1997). Anorexia of aging: physiologic and pathologic. Am. J. Clin. Nutr. 66, 760–773 9322549 10.1093/ajcn/66.4.760

[B104] Morley J. E. Baumgartner R. N. Roubenoff R. Mayer J. Nair K. S. (2001). Sarcopenia. J. Lab. Clin. Med. 137, 231–243 10.1067/mlc.2001.113504 11283518

[B105] Morse C. I. Thom J. M. Mian O. S. Muirhead A. Birch K. M. Narici M. V. (2005). Muscle strength, volume and activation following 12-month resistance training in 70-year-old males. Eur. J. Appl. Physiol. 95, 197–204 10.1007/s00421-005-1342-3 16003538

[B106] Muller-Delp J. M. Spier S. A. Ramsey M. W. Delp M. D. (2002). Aging impairs endothelium-dependent vasodilatation in rat skeletal muscle arterioles. Am. J. Physiol. Heart Circ. Physiol. 283, H1662–H1672 12234821 10.1152/ajpheart.00004.2002

[B107] Narici M. V. Maffulli N. (2010). Sarcopenia: characteristics, mechanisms and functional significance. Br. Med. Bull. 95, 139–159 10.1093/bmb/ldq008 20200012

[B108] Narici M. V. Maganaris C. N. (2007). Plasticity of the muscle-tendon complex with disuse and aging. Exerc. Sport Sci. Rev. 35, 126–134 17620931 10.1097/jes.0b013e3180a030ec

[B109] Narici M. V. Maganaris C. N. Reeves N. D. Capodaglio P. (2003). Effect of aging on human muscle architecture. J. Appl. Physiol. 95, 2229–2234 12844499 10.1152/japplphysiol.00433.2003

[B110] National Institutes of Health N. (2004). Exercise: A Guide from the National Institute on Aging. Bethesda: U.S. Department of Health and Human Services

[B111] Newman A. B. Kupelian V. Visser M. Simonsick E. M. Goodpaster B. H. Kritchevsky S. B. Tylavsky F. A. Rubin S. M. Harris T. B. (2006). Strength, but not muscle mass, is associated with mortality in the health, aging and body composition study cohort. J. Gerontol. A Biol. Sci. Med. Sci. 61, 72–77 10.1093/gerona/61.1.72 16456196

[B112] Novak L. P. (1972). Aging, total body potassium, fat-free mass, and cell mass in males and females between ages 18 and 85 years. J. Gerontol. 27, 438–443 4627605 10.1093/geronj/27.4.438

[B113] Nygaard E. Sanchez J. (1982). Intramuscular variation of fiber types in the brachial biceps and the lateral vastus muscles of elderly men: how representative is a small biopsy sample? Anat. Rec. 203, 451–459 10.1002/ar.1092030404 7137599

[B114] Office of Fair Trading O. (2005). Care Homes for Older People in the UK – A Market Study. OFT780, OFT780. Available at: www.oft.gov.uk/shared_oft/reports/consumer_protection/oft780.pdf

[B115] Ojanen T. Rauhala T. Hakkinen K. (2007). Strength and power profiles of the lower and upper extremities in master throwers at different ages. J. Strength Cond. Res. 21, 216–222 10.1519/00124278-200702000-00039 17313300

[B116] Peake J. Della Gatta P. Cameron-Smith D. (2010). Aging and its effects on inflammation in skeletal muscle at rest and following exercise-induced muscle injury. Am. J. Physiol. Regul. Integr. Comp. Physiol. 298, R1485–R1495 10.1152/ajpregu.00467.2009 20393160

[B117] Pedersen M. Bruunsgaard H. Weis N. Hendel H. W. Andreassen B. U. Eldrup E. Dela F. Pedersen B. K. (2003). Circulating levels of TNF-alpha and IL-6-relation to truncal fat mass and muscle mass in healthy elderly individuals and in patients with type-2 diabetes. Mech. Ageing Dev. 124, 495–502 10.1016/S0047-6374(03)00027-7 12714258

[B118] Percheron G. Hogrel J. Y. Denot-Ledunois S. Fayet G. Forette F. Baulieu E. E. Fardeau M. Marini J. F. (2003). Effect of 1-year oral administration of dehydroepiandrosterone to 60- to 80-year-old individuals on muscle function and cross-sectional area: a double-blind placebo-controlled trial. Arch. Intern. Med. 163, 720–727 10.1001/archinte.163.6.720 12639206

[B119] Phillips S. K. Woledge R. C. Bruce S. A. Young A. Levy D. Yeo A. Martin F. C. (1998). A study of force and cross-sectional area of adductor pollicis muscle in female hip fracture patients. J. Am. Geriatr. Soc. 46, 999–1002 9706890 10.1111/j.1532-5415.1998.tb02756.x

[B120] Porter M. M. Vandervoort A. A. Kramer J. F. (1997). Eccentric peak torque of the plantar and dorsiflexors is maintained in older women. J. Gerontol. A Biol. Sci. Med. Sci. 52, B125–B131 10.1093/gerona/52A.2.B125 9060970

[B121] Pousson M. Lepers R. Van Hoecke J. (2001). Changes in isokinetic torque and muscular activity of elbow flexors muscles with age. Exp. Gerontol. 36, 1687–1698 10.1016/S0531-5565(01)00143-7 11672989

[B122] Power G. A. Dalton B. H. Behm D. G. Doherty T. J. Vandervoort A. A. Rice C. L. (2012). Motor unit survival in life-long runners is muscle-dependent. Med. Sci. Sports Exerc. 44, 1235–1242 22246219 10.1249/MSS.0b013e318249953c

[B123] Prior S. J. Roth S. M. Wang X. Kammerer C. Miljkovic-Gacic I. Bunker C. H. Wheeler V. W. Patrick A. L. Zmuda J. M. (2007). Genetic and environmental influences on skeletal muscle phenotypes as a function of age and sex in large, multigenerational families of African heritage. J. Appl. Physiol. 103, 1121–1127 10.1152/japplphysiol.00120.2007 17656630 PMC2811418

[B124] Proctor D. N. Shen P. H. Dietz N. M. Eickhoff T. J. Lawler L. A. Ebersold E. J. Loeffler D. L. Joyner M. J. (1998). Reduced leg blood flow during dynamic exercise in older endurance-trained men. J. Appl. Physiol. 85, 68–75 9655757 10.1152/jappl.1998.85.1.68

[B125] Purves D. Augustine G. Fitzpatrick D. Katz L. C. Lamantia A. S. Mcnamara J. O. Williams S. M. (ed.). (2001). Neurosciences. Sunderland, MA: Sinauer Associates

[B126] Ratkevicius A. Joyson A. Selmer I. Dhanani T. Grierson C. Tommasi A. M. Devries A. Rauchhaus P. Crowther D. Alesci S. Yaworsky P. Gilbert F. Redpath T. W. Brady J. Fearon K. C. Reid D. M. Greig C. A. Wackerhage H. (2011). Serum concentrations of myostatin and myostatin-interacting proteins do not differ between young and sarcopenic elderly men. J. Gerontol. A Biol. Sci. Med. Sci. 66, 620–626 10.1093/gerona/glr025 21382886

[B127] Raue U. Slivka D. Minchev K. Trappe S. (2009). Improvements in whole muscle and myocellular function are limited with high-intensity resistance training in octogenarian women. J. Appl. Physiol. 106, 1611–1617 10.1152/japplphysiol.91587.2008 19246651 PMC2681332

[B128] Roberts S. B. (1995). Effects of aging on energy requirements and the control of food intake in men. J. Gerontol. A Biol. Sci. Med. Sci. 50, 101–106 7493200 10.1093/gerona/50a.special_issue.101

[B129] Roig M. Macintyre D. L. Eng J. J. Narici M. V. Maganaris C. N. Reid W. D. (2010). Preservation of eccentric strength in older adults: evidence, mechanisms and implications for training and rehabilitation. Exp. Gerontol. 45, 400–409 10.1016/j.exger.2010.03.008 20303404 PMC3326066

[B130] Rolland Y. Czerwinski S. Abellan Van Kan G. Morley J. E. Cesari M. Onder G. Woo J. Baumgartner R. Pillard F. Boirie Y. Chumlea W. M. Vellas B. (2008). Sarcopenia: its assessment, etiology, pathogenesis, consequences and future perspectives. J. Nutr. Health Aging 12, 433–450 10.1007/BF02982704 18615225 PMC3988678

[B131] Rosenberg I. (1989). Summary comments. Am. J. Clin. Nutr. 50, 1231–1233

[B132] Roubenoff R. Harris T. B. Abad L. W. Wilson P. W. Dallal G. E. Dinarello C. A. (1998). Monocyte cytokine production in an elderly population: effect of age and inflammation. J. Gerontol. A Biol. Sci. Med. Sci. 53, M20–M26 10.1093/gerona/53A.1.M20 9467429

[B133] Roubenoff R. Hughes V. A. (2000). Sarcopenia: current concepts. J. Gerontol. A Biol. Sci. Med. Sci. 55, M716–M724 10.1093/gerona/55.12.M757 11129393

[B134] Rudman D. Feller A. G. Nagraj H. S. Gergans G. A. Lalitha P. Y. Goldberg A. F. Schlenker R. A. Cohn L. Rudman I. W. Mattson D. E. (1990). Effects of human growth hormone in men over 60 years old. N. Engl. J. Med. 323, 1–6 10.1056/NEJM199007053230101 2355952

[B135] Sayhoun N. (1992). “Nutrient intake by the NSS elderly population,” in Nutrition in the Elderly: The Boston Nutritional Survey, eds Hartz S. R. Russell R. M. Rosenbeerg I. H. (London: Smith-Gordon and Company).

[B136] Sehl M. E. (2001). Senescence, frailty and mortality: mathematical models of aging. Med. Health RI 84, 360–364 12355663

[B137] Seibert M. J. Xue Q. L. Fried L. P. Walston J. D. (2001). Polymorphic variation in the human myostatin (GDF-8) gene and association with strength measures in the Women’s Health and Aging Study II cohort. J. Am. Geriatr. Soc. 49, 1093–1096 10.1046/j.1532-5415.2001.49214.x 11555072

[B138] Silva A. M. Shen W. Heo M. Gallagher D. Wang Z. Sardinha L. B. Heymsfield S. B. (2009). Ethnicity-related skeletal muscle differences across the lifespan. Am. J. Hum. Biol. 22, 76–82 10.1002/ajhb.20956 19533617 PMC2795070

[B139] Siriett V. Platt L. Salerno M. S. Ling N. Kambadur R. Sharma M. (2006). Prolonged absence of myostatin reduces sarcopenia. J. Cell. Physiol. 209, 866–873 10.1002/jcp.20778 16972257

[B140] Sirola J. Kroger H. (2011). Similarities in acquired factors related to postmenopausal osteoporosis and sarcopenia. J. Osteoporos. 2011, 536735 21904688 10.4061/2011/536735 PMC3166567

[B141] Skelton D. A. Greig C. A. Davies J. M. Young A. (1994). Strength, power and related functional ability of healthy people aged 65–89 years. Age Ageing 23, 371–377 10.1093/ageing/23.5.371 7825481

[B142] Solerte S. B. Gazzaruso C. Bonacasa R. Rondanelli M. Zamboni M. Basso C. Locatelli E. Schifino N. Giustina A. Fioravanti M. (2008). Nutritional supplements with oral amino acid mixtures increases whole-body lean mass and insulin sensitivity in elderly subjects with sarcopenia. Am. J. Cardiol. 101, 69e–77e 10.1016/j.amjcard.2007.07.050 18514630

[B143] Sweeney H. L. Bowman B. F. Stull J. T. (1993). Myosin light chain phosphorylation in vertebrate striated muscle: regulation and function. Am. J. Physiol. 264, C1085–C1095 8388631 10.1152/ajpcell.1993.264.5.C1085

[B144] Taekema D. G. Gussekloo J. Maier A. B. Westendorp R. G. de Craen A. J. (2010). Handgrip strength as a predictor of functional, psychological and social health. A prospective population-based study among the oldest old. Age Ageing 39, 331–337 10.1093/ageing/afq022 20219767

[B145] Tan L. J. Liu S. L. Lei S. F. Papasian C. J. Deng H. W. (2012). Molecular genetic studies of gene identification for sarcopenia. Hum. Genet. 131, 1–31 10.1007/s00439-011-1040-7 21706341

[B146] Tanko L. B. Movsesyan L. Mouritzen U. Christiansen C. Svendsen O. L. (2002). Appendicular lean tissue mass and the prevalence of sarcopenia among healthy women. Metab. Clin. Exp. 51, 69–74 10.1053/meta.2002.28960 11782875

[B147] Thom J. M. Morse C. I. Birch K. M. Narici M. V. (2007). Influence of muscle architecture on the torque and power-velocity characteristics of young and elderly men. Eur. J. Appl. Physiol. 100, 613–619 10.1007/s00421-007-0481-0 17530274

[B148] Thomas D. R. (2007). Loss of skeletal muscle mass in aging: examining the relationship of starvation, sarcopenia and cachexia. Clin. Nutr. 26, 389–399 10.1016/j.clnu.2007.03.008 17499396

[B149] Tomlinson B. E. Irving D. (1977). The numbers of limb motor neurons in the human lumbosacral cord throughout life. J. Neurol. Sci. 34, 213–219 10.1016/0022-510X(77)90069-7 925710

[B150] Tomlinson B. E. Walton J. N. Rebeiz J. J. (1969). The effects of ageing and of cachexia upon skeletal muscle. A histopathological study. J. Neurol. Sci. 9, 321–346 10.1016/0022-510X(69)90079-3 4899241

[B151] Tzankoff S. P. Norris A. H. (1977). Effect of muscle mass decrease on age-related BMR changes. J. Appl. Physiol. 43, 1001–1006 606683 10.1152/jappl.1977.43.6.1001

[B152] Urban R. J. Bodenburg Y. H. Gilkison C. Foxworth J. Coggan A. R. Wolfe R. R. Ferrando A. (1995). Testosterone administration to elderly men increases skeletal muscle strength and protein synthesis. Am. J. Physiol. 269, E820–E86 7491931 10.1152/ajpendo.1995.269.5.E820

[B153] Visser M. Deeg D. J. Lips P. (2003). Low vitamin D and high parathyroid hormone levels as determinants of loss of muscle strength and muscle mass (sarcopenia): the Longitudinal Aging Study Amsterdam. J. Clin. Endocrinol. Metab. 88, 5766–5772 10.1210/jc.2003-030604 14671166

[B154] Visser M. Goodpaster B. H. Kritchevsky S. B. Newman A. B. Nevitt M. Rubin S. M. Simonsick E. M. Harris T. B. (2005). Muscle mass, muscle strength, and muscle fat infiltration as predictors of incident mobility limitations in well-functioning older persons. J. Gerontol. A Biol. Sci. Med. Sci. 60, 324–333 10.1093/gerona/60.3.324 15860469

[B155] Visser M. Harris T. B. Langlois J. Hannan M. T. Roubenoff R. Felson D. T. Wilson P. W. Kiel D. P. (1998a). Body fat and skeletal muscle mass in relation to physical disability in very old men and women of the Framingham Heart Study. J. Gerontol. A Biol. Sci. Med. Sci. 53, M214–M221 10.1093/gerona/53A.3.M214 9597054

[B156] Visser M. Langlois J. Guralnik J. M. Cauley J. A. Kronmal R. A. Robbins J. Williamson J. D. Harris T. B. (1998b). High body fatness, but not low fat-free mass, predicts disability in older men and women: the Cardiovascular Health Study. Am. J. Clin. Nutr. 68, 584–590 9734734 10.1093/ajcn/68.3.584

[B157] Volpi E. Nazemi R. Fujita S. (2004). Muscle tissue changes with aging. Curr. Opin. Clin. Nutr. Metab. Care 7, 405–410 10.1097/01.mco.0000134362.76653.b2 15192443 PMC2804956

[B158] Wang Z. M. Visser M. Ma R. Baumgartner R. N. Kotler D. Gallagher D. Heymsfield S. B. (1996). Skeletal muscle mass: evaluation of neutron activation and dual-energy X-ray absorptiometry methods. J. Appl. Physiol. 80, 824–831 8964743 10.1152/jappl.1996.80.3.824

[B159] Whittemore L. A. Song K. Li X. Aghajanian J. Davies M. Girgenrath S. Hill J. J. Jalenak M. Kelley P. Knight A. Maylor R. O’Hara D. Pearson A. Quazi A. Ryerson S. Tan X. Y. Tomkinson K. N. Veldman G. M. Widom A. Wright J. F. Wudyka S. Zhao L. Wolfman N. M. (2003). Inhibition of myostatin in adult mice increases skeletal muscle mass and strength. Biochem. Biophys. Res. Commun. 300, 965–971 10.1016/S0006-291X(02)02953-4 12559968

[B160] Wilkes E. A. Selby A. L. Atherton P. J. Patel R. Rankin D. Smith K. Rennie M. J. (2009). Blunting of insulin inhibition of proteolysis in legs of older subjects may contribute to age-related sarcopenia. Am. J. Clin. Nutr. 90, 1343–1350 10.3945/ajcn.2009.27543 19740975

[B161] Wroblewski A. P. Amati F. Smiley M. A. Goodpaster B. Wright V. (2011). Chronic exercise preserves lean muscle mass in masters athletes. Phys. Sportsmed. 39, 172–178 10.3810/psm.2011.09.1933 22030953

[B162] Yende S. Waterer G. W. Tolley E. A. Newman A. B. Bauer D. C. Taaffe D. R. Jensen R. Crapo R. Rubin S. Nevitt M. Simonsick E. M. Satterfield S. Harris T. Kritchevsky S. B. (2006). Inflammatory markers are associated with ventilatory limitation and muscle dysfunction in obstructive lung disease in well functioning elderly subjects. Thorax 61, 10–16 10.1136/thx.2004.034181 16284220 PMC2080698

[B163] Young A. Stokes M. Crowe M. (1985). The size and strength of the quadriceps muscles of old and young men. Clin. Physiol. 5, 145–154 10.1111/j.1475-097X.1985.tb00590.x 3888498

[B164] Yu F. Hedstrom M. Cristea A. Dalen N. Larsson L. (2007). Effects of ageing and gender on contractile properties in human skeletal muscle and single fibres. Acta Physiol. (Oxf.) 190, 229–241 10.1111/j.1748-1716.2007.01699.x 17581136

